# Locomotion-dependent use of geometric and body cues in humans mapping 3D space

**DOI:** 10.1073/pnas.2505613122

**Published:** 2025-12-19

**Authors:** Volker Reisner, Theo A. J. Schäfer, Leonard König, Misun Kim, Christian F. Doeller

**Affiliations:** ^a^Department of Psychology, Max Planck Institute for Human Cognitive and Brain Sciences, Leipzig 04103, Germany; ^b^German Center for Neurodegenerative Diseases, Magdeburg 39120, Germany; ^c^Institute of Psychology, University of Hamburg, Hamburg 20146, Germany; ^d^Institute of Cognitive Neuroscience, University College London, London WC1N 3AZ, United Kingdom; ^e^Kavli Institute for Systems Neuroscience, Centre for Neural Computation, The Egil and Pauline Braathen and Fred Kavli Centre for Cortical Microcircuits, Jebsen Centre for Alzheimer’s Disease, Norwegian University of Science and Technology, Trondheim 7491, Norway

**Keywords:** cognitive map, 3D space, environmental geometry, spatial memory, virtual reality

## Abstract

The ability to represent locations across multiple dimensions of space is a core function of cognitive maps. While the influence of boundary-dependent environmental geometry on spatial representations has been extensively studied in 2D spaces, less is known about the role of boundaries for volumetric spatial memory. Research in humans and other animals has demonstrated distinct processing of the vertical and horizontal spatial dimensions, likely related to species-specific modes of locomotion. Here, we investigate whether different locomotion modes, flying and walking, affect the use of vertical boundaries, leading to possibly distinct volumetric representations. In a Virtual Reality experiment, human participants memorized objects within a symmetric 3D enclosure, and then were asked to replace them in either the familiar or geometrically deformed environments. We found that the flying group exhibited lower vertical than horizontal spatial memory precision, whereas the walking group showed the opposite pattern, an effect related to using their body axis as a vertical “ruler”. Within deformed environments, object replacements in the flying group followed the predictions from a 3D-extended boundary-vector-cell-like computational model of spatial mapping that treated all boundaries equally, whereas those in the walking condition favored a modified model that prioritized the ground boundary. Our findings suggest that gravity-related movement constraints promote different utilization of geometric and body-related cues, resulting in flexible representations of volumetric space.

## Significance

Environmental boundaries provide essential anchors for spatial navigation, yet little is known about how vertical and horizontal boundaries influence spatial representations in the 3D space. Using immersive Virtual Reality, we show that boundary-based encoding depends on how we move in 3D space. Virtually flying participants relied on boundary information uniformly across three dimensions, aligning with a computational model inspired by spatially tuned neurons. In contrast, walking participants prioritized the ground as an anchoring reference and used their body axis as a vertical “ruler”, resulting in greater vertical spatial precision. These findings highlight how gravity-related constraints and environmental geometry reconfigure human cognitive maps allowing for flexible navigation in a 3D world.

Representing locations in space constitutes a key function of the cognitive map, a mental model of the environment that supports flexible navigation (1–2). Extensive research across species has demonstrated that cognitive maps are significantly influenced by environmental boundaries. These serve as highly stable reference cues, which separate space into navigable regions and define their geometric shape (3–13). For instance, when the geometry of a familiar enclosure was changed from a square to a rectangle, both the firing patterns of rodent place cells and human spatial memory responses adapted according to the geometric properties of the new environment (7, 12). In the brain, these properties are signaled by boundary-vector cells (BVCs), each firing at a specific allocentric direction and distance from the walls (14, 15). Notably, computational models mimicking the population activity of BVCs have successfully predicted both rodent place cell activity and human spatial memory in geometrically deformed environments (7, 12, 16–18), suggesting a common neural mechanism for anchoring cognitive maps across species (19, 20).

How boundary information is utilized and encoded during navigation has mostly been studied on horizontal surfaces. However, while animals routinely traverse three-dimensional environments, little is known about how vertical boundaries, such as floors and ceilings, contribute to the formation of three-dimensional cognitive maps. Understanding the coding principles of the third spatial dimension is crucial for expanding our knowledge of how multidimensional cognitive maps support broader aspects of cognition, extending beyond physical navigation (21, 22).

The vertical spatial dimension is unique due to the influence of gravity. Flying animals can more readily defy gravity and approach boundaries along both horizontal and vertical dimensions to estimate distance. Place cell recordings in freely flying bats exhibited isotropic firing fields with similar resolution across spatial dimensions (23). In contrast, surface-dwelling animals are more constrained in their movements toward boundaries in the opposite direction of gravity (ceiling) and may need to rely more on vision for vertical distance estimation. However, depth perception along the vertical axis can be less accurate due to head tilt (24, 25), which may contribute to anisotropic spatial representations observed in both rats (26, 27) and humans (28–30) [for diverging findings see (31, 32)]. Given these cross-species differences, it was hypothesized that surface-dwelling animals such as ourselves, might have evolved to prioritize horizontal over vertical spatial information due to their natural mode of locomotion (33).

Nonetheless, gravity can also serve as a useful cue for representing vertical space relative to the ground. This becomes particularly clear in environments with reduced gravity, like on space stations, where astronauts often report severe disorientation (34). On earth, gravity ensures stable sensory feedback from the vestibular system which, in primates, can signal tilt along the vertical dimension and is not available for horizontal directions (35). At the same time, gravity consistently defines a stable vertical axis which is aligned with the upright human body axis that rises from the ground. When moving on a horizontal plane, the body’s orthogonal relationship to the ground remains constant, providing a reliable point of reference and, thus, potentially making it easier to estimate height between the ground and one’s own body dimensions (e.g., the head).

In the present study, we sought to understand the role of environmental boundaries in the representation of three-dimensional space, considering two locomotion modes: one that allows for 3D movement relatively unconstrained by gravity, similar to winged animals (flying group), and one that is restricted to the ground, reflecting naturalistic human locomotion under gravity’s influence (walking group). This allowed us to investigate how humans integrate environmental boundary cues and gravity-dependent, body-based cues under different degrees of freedom for moving against gravity. Furthermore, we deformed the geometry of the environment to investigate the causal influence of boundaries on spatial memory. By applying computational geometric models (7) extended into 3D space, we uncovered distinct strategies depending on the mode of locomotion: those who walked tended to rely more on ground- and body-based references for vertical estimation, while those who flew exhibited more equal encoding relative to boundaries across dimensions.

## Results

**Fig. 1.**
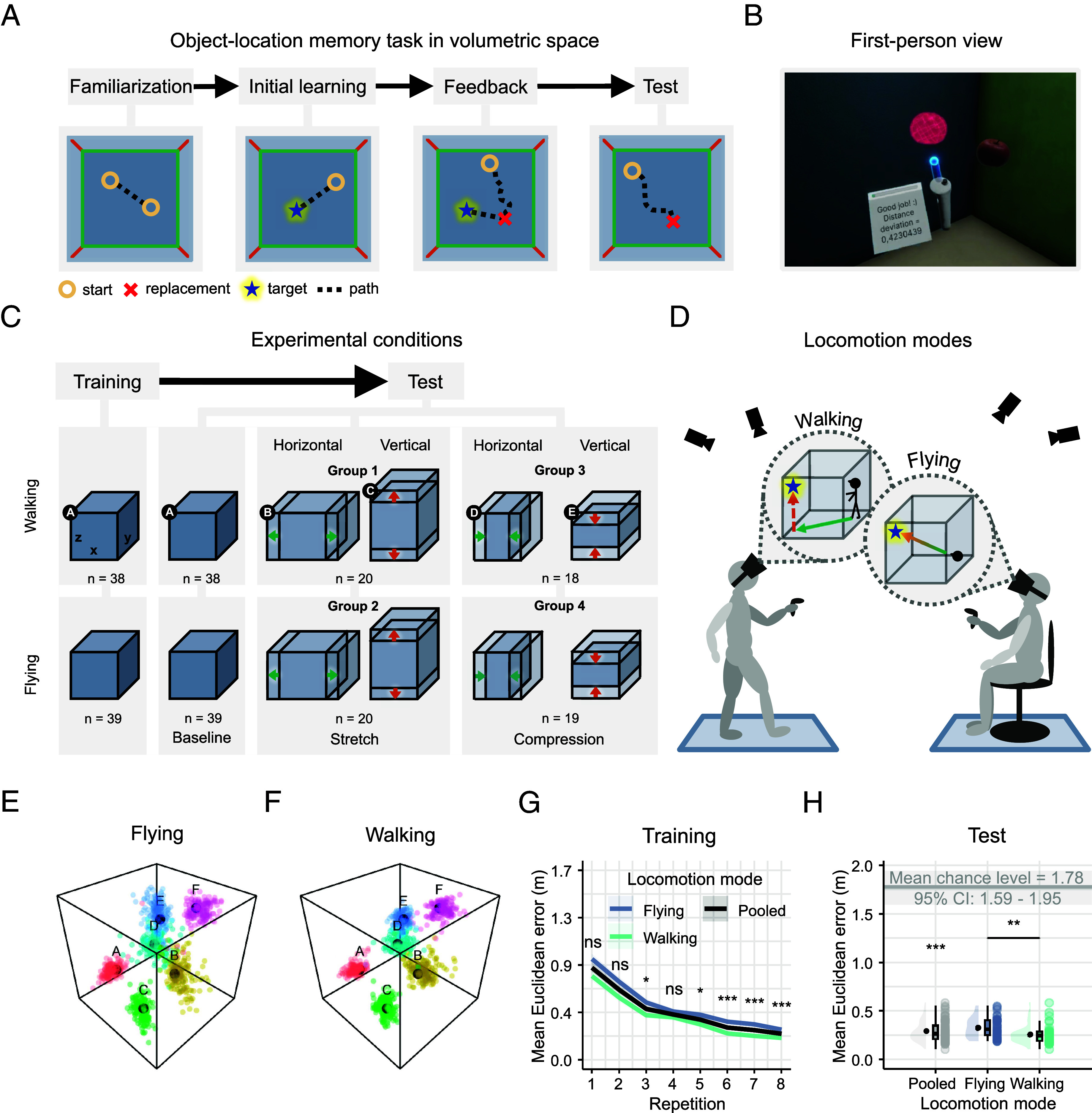
Experimental task and design. (*A*) Object-location memory task in volumetric space. Familiarization: Participants practiced navigating (dotted line) within the 3D virtual environment by collecting freely floating balls (circles) in succession. Initial learning: Participants memorized virtual everyday-life objects at six target locations, fixed in volumetric space (star), always starting from random locations (indicated by a ball). Feedback: Participants replaced each object at its corresponding target location from memory (cross), starting from a ball’s random location. After each response, they received immediate feedback about their accuracy and re-encoded the object’s correct location before the beginning of the next trial. Test: Participants continued replacing objects at remembered target locations but without receiving feedback. (*B*) First-person view during feedback. Feedback was provided based on the displacement of the object replacement from the target location in meters. The true target location was displayed alongside the corresponding replacement location (red hologram). (*C*) During training, object-locations were learned in the baseline environment (cube). During the test, spatial memory was assessed in both the baseline and in the deformed environments (environments A-E). Conditions were manipulated in a 2 × 2 × 2 mixed design with the within-subject factor environmental deformation (horizontal vs vertical plane) and the between-subject factors type of deformation (stretched vs compressed) and mode of locomotion (flying vs walking). The baseline environment (3 × 3 × 3 m) was deformed by a factor of 1.33 (33%) for horizontal or vertical dimensions (*SI Appendix*, Table S2). Note that all participants were naive about the deformations. (*D*) We divided participants into two groups with distinct locomotion modes, differing relatively in their constraint by gravity. In the walking group, participants freely moved on the horizontal surface of the enclosure, constrained by gravity, and they reached vertical positions by extending their arm or a virtual wand. In the flying group, participants virtually navigated in all three dimensions, relatively unconstrained by gravity, while physically sitting on a rotatable chair. Translation movement (forward/backward) was achieved via the controller, and flying direction (pitch × yaw) was set by their head rotation. Motion-capture cameras recorded the movements of a set of rigid bodies attached to participants’ body parts (*SI Appendix*, Fig. S1*B*). (*E* and *F*) Replacement locations (color-coded) and the true target locations (labeled black dots), separately for the walking (*E*) and flying (*F*) group in the baseline condition. Each dot represents a single trial object replacement of a given participant during the test phase. (*G*) Baseline training performance (Euclidean error in 3D space) as a function of repetition, averaged across target locations, separately for each locomotion mode and pooled (color-coded lines depict means ± SEM). Mean spatial memory error (in meters) decreased over repetitions. Flying participants showed higher errors than walking participants in the last few trials. (*H*) Baseline test performance (Euclidean error in 3D space) for each locomotion mode and pooled. The mean spatial memory error was significantly lower than the mean chance level (gray horizontal line) with flying participants showing higher errors than walking participants. Violin plots depict the density distribution, boxplots the median and quartiles, black dots with error bars the means ± SEM, and colored dots individual data points per condition. *P < 0.05, **P < 0.01, ***P < 0.001.

We analyzed data from 77 healthy young participants (40 female, 36 male, 1 nonbinary; Age: 26.2 ± 4.5 y, 19 to 35 y) performing a 3D object-location memory task in an immersive Virtual Reality (VR) with Motion Capture (MoCap) technology (Fig. 1 *A* and *B*). During training, participants first learned 3D locations of 6 free-floating objects within a cubic baseline environment. This manipulation is different from previous studies where objects were lying on the walls, floor, or discretized 3D structure (28, 29, 31, 32). During the subsequent test, participants were asked to replace the objects from pseudorandom start locations. We probed their volumetric spatial memory in either the familiar or geometrically deformed environments (Fig. 1*C*). While for some participants the deformed environment was stretched (groups 1 and 2) and for others it was compressed (groups 3 & 4), all participants encountered deformations along both the horizontal and vertical dimensions. Ultimately, participants completed the experiment in one of two locomotion modes, with walking participants (groups 1 & 3) performing natural human locomotion and virtual flying participants mimicking the natural locomotion of winged animals (groups 2 and 4; Fig. 1*D*). The full experimental design is shown in Fig. 1*C*.

### Successful Encoding of 3D Coordinates in Volumetric Space.

Previous research in humans investigated spatial memory in 3D space exclusively by probing target locations projected onto surfaces or within discrete spaces. But can human participants sufficiently encode object-locations in fully volumetric space? To test this, we assessed participants’ spatial memory accuracy during training and test (environment A in Fig. 1*C*; Fig. 1 *E* and *F*). For each trial, we measured the distance between the object-replacement and the correct target location in 3D Euclidean space indicating a mnemonic error in representing object-locations. During training, spatial memory errors overall decreased as a function of replacement repetition (1-way repeated-measures ANOVA: *F*_2.93,222.3_ = 122.76, *P* < 0.001, $\eta_{p}^{2}$ = 0.618; Fig. 1*G*) with an average improvement of 0.6 m from the first to the last repetition (post hoc paired *t* test: *t*_76_ = 15.3, *P*_adj_ < 0.001, *d* = 1.74; Bonferroni-corrected for 28 comparisons). During test (when no feedback was provided), spatial memory accuracy remained high (M = 0.30 m, SD = 0.10 m), significantly falling below the mean chance level based on simulated random errors (M = 1.77 m, SD = 0.24 m, bootstrapped 95% CI = 1.60 to 1.94 m; 1-sample *t* test: *t*_76_ = −127, *P* < 0.001, *d* = −14.4; Fig. 1*H*), demonstrating that participants successfully encoded freely floating objects in volumetric space. However, when comparing spatial memory performance across different modes of locomotion, we found that flying participants exhibited overall higher errors than walking participants, both at training (one-way ANOVA: *F*_1,614_ = 20.811, *P* < 0.001, $\eta_{p}^{2}$ = 0.033; Fig. 1*G*) and at test (two-sample *t* test: *t*_73.4_ = 3.07, *P* = 0.003, *d* = 0.7; Fig. 1*H*).

### Anisotropic Representation of Volumetric Space Is Locomotion-Dependent.

**Fig. 2.**
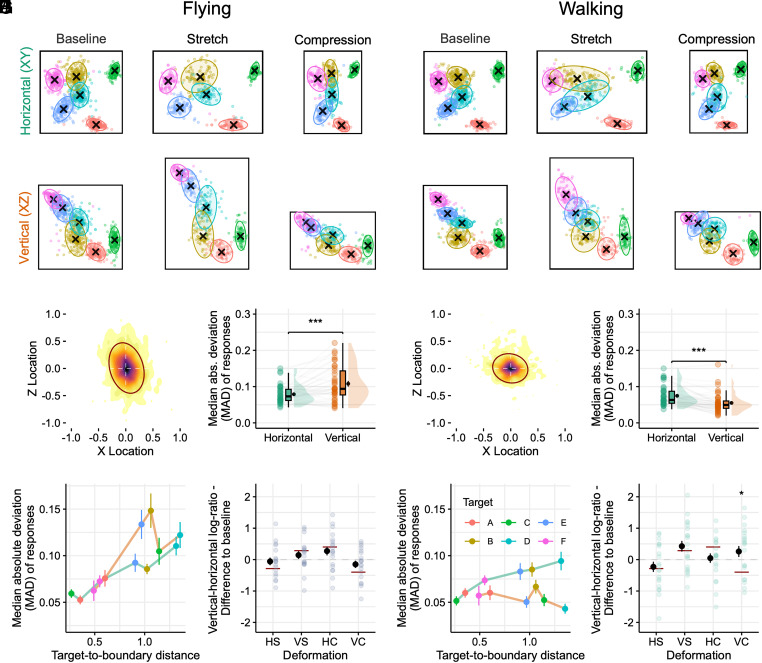
Locomotion-dependent anisotropy in the representation of volumetric space. (*A*, *C*, *D*, *G*, and *H*) Flying group. (*B*, *E*, *F*, *I*, and *J*) Walking group. (*A* and *B*) Trial-wise object replacements projected onto 2D planes, deformation type (columns) and deformation axes (rows). Ellipses refer to areas including 95% of responses for different target locations (color-coded) with black crosses demarking their median. (*C* and *E*) Distribution of median-centered replacement responses in the baseline testing environment, pooled over target locations, reveals larger dispersion along the z-axis in the flying condition (*C*) and larger dispersion along the x-axis in the walking condition (*E*). Darker colors reflect higher frequency of values. (*D* and *F*) Median absolute deviation (MAD in meters) in the baseline for the horizontal (x-, y-axis) and vertical (z-axis) for the flying (*D*) and walking (*F*) groups. Violin plots depict the density distribution, boxplots the median and quartiles, mean ± SEM as black dots with error bars, as well as individual data points (dots) per condition. ***P < 0.001. (*G* and *I*) MAD for different target locations as a function of target-to-boundary distance, separately computed for the horizontal dimension (green line) and vertical dimension (orange line). While the flying group (*G*) shows a positive distance–MAD relationship in both dimensions, the walking group (*I*) only shows this relationship in the horizontal dimension. (*H* and *J*) Vertical anisotropy indices defined as vertical to horizontal MAD log-ratio for different deformation types (HS/VS: Horizontally/vertically stretched, HC/VC: Horizontally/vertically compressed) relative to baseline. Observed anisotropy indices (dots ± SE) were tested against the expected anisotropy change induced by the 33% deformation (red bars), computed as the log-ratio of the vertical and horizontal deformation size: ln(V/H); VS: ln(1.33/1) = +0.285; HC: ln(1/0.67) = +0.400; HS: ln(1/1.33) = −0.285; VC: ln(0.67/1) = −0.400. In the VC condition, the walking group differed significantly from the expected elongation. *P < 0.05 (Bonferroni-corrected for 8 comparisons).

To evaluate whether humans memorize volumetric space equally across spatial dimensions and how this might be influenced by the mode of locomotion, we analyzed dimensional differences in participants’ response dispersion. To this end, responses (for target-specific responses see Fig. 2 *A* and *B* and *SI Appendix*, Fig. S2) were pooled and median-centered across target locations for each group (Fig. 2 *C* and *E*), and their median absolute deviation (MAD) was computed for each dimension (Fig. 2 *D* and *F*). A significant interaction between spatial dimension and locomotion mode was found in the baseline environment (two-way mixed ANOVA: *F*_1,75_ = 40.73, *P* < 0.001, $\eta_{p}^{2}$ = 0.352), with a higher response dispersion along the vertical compared to the horizontal dimension in the flying group (paired *t* test: *t*_36_ = 4.34, *P* < 0.001, *d* = 0.713, Bonferroni-corrected for 2 comparisons; Fig. 2*D*). A contrary effect was observed in the walking group (*t*_39_ = −4.99, *P* < 0.001, *d* = −0.789; Fig. 2*F*), suggesting a lower vertical precision for flying participants and a higher vertical precision for walking participants. Notably, we found a significant difference between the flying and walking groups only in the vertical dimension (*t*_55.8_ = 5.88, *P* < 0.001, *d* = 1.36), but not in the horizontal dimensions (*t*_75_ = 0.698, *P* = 0.974, *d* = 0.159), indicating that only vertical information is represented differently depending on the mode of locomotion.

Next, we examined whether differences in navigational behavior between locomotion modes underlie the observed differences in vertical processing. We found that participants of the walking group visited fewer locations in the volumetric space compared to the flying group (2-sample *t* test: *t*_74.5_ = 3.38, *P* = 0.001, *d* = 0.771; *SI Appendix*, Fig. S4*C*) and their movement paths between the start and replacement location were shorter (*t*_71.8_ = −2.24, *P* = 0.028, *d* = −0.508), indicating higher confidence in spatial memory. Their location-specific movement paths were also much more similar across trials (*t*_61.7_ = 10.1, *P* < 0.001, *d* = 2.32), suggesting stronger reliance on route-based strategies. Moreover, their head orientation at replacement during test differed less from the orientation at collection during training than in the flying condition (*t*_52.4_ = 3.57, *P* < 0.001, *d* = 0.823), indicating a stronger tendency for perceptual matching. Only heading difference (Spearman’s *Rho* = 0.52, *S* = 36594, *P* < 0.001; *SI Appendix*, Fig. S4*D*) and path dissimilarity (Spearman’s *Rho* = 0.66, *S* = 25672, *P* < 0.001; all other *P* > 0.126) correlated with vertical dispersion. Together, these results highlight the role of the body serving as an egocentric reference frame during naturalistic, bipedal walking that can shape the vertical-horizontal symmetry of human spatial memory.

### Distinct Use of Vertical Boundaries and Body Cues for Height Estimation During Naturalistic Walking.

In 2D rectangular environments, locations are encoded relative to the enclosure’s boundaries, resulting in better memory for locations close to boundaries than far from them (7, 19). To evaluate whether the same relation holds for both horizontal and vertical boundaries in each locomotion mode, we computed the correlation between the response dispersion and the distance to the nearest boundary for each dimension (Fig. 2 *G* and *I*). As expected, both locomotion groups exhibited a comparable positive relationship between the response dispersion and the target-to-boundary distance in the horizontal dimension (walking: average Spearman’s *Rho* = 0.33, *t*_39_ = 4.84, *P* < 0.001; flying: Spearman’s *Rho* = 0.481, *t*_36_ = 7.78, *P* < 0.001). However, the two groups differed significantly with respect to the vertical dimension. Flying participants showed a positive (Spearman’s *Rho* = 0.327, *t*_36_ = 5.27, *P* < 0.001; Fig. 2*G*) and walking participants no relationship (Spearman’s *Rho* = −0.0429, *t*_39_ = −0.551, *P* = 0.584; Fig. 2*I*).

One possible explanation for why walking participants might process vertical spatial information differently from flying participants is that, in addition to relying on environmental boundaries, they could encode locations relative to their upright body. This posture is reliably anchored to the ground plane of locomotion and aligned with gravity. Thus, locations near the vertical center are not only farthest from vertical boundaries but also closest to the participant’s head height (participant height: M = 1.74 m, SD = 0.07 m; height of object-location D: 1.65 m). Indeed, we found increased spatial memory dispersion as the distance between the height of a target location and the height of the walking participant’s head increased (1-sample *t* test on individual correlations between MAD and target-to-head distance: Average Spearman’s *Rho* = 0.18, *t*_39_ = 2.26, *P* = 0.029). This suggests that both vertical boundaries and the participants’ own bodies, particularly the position of their heads, serve as useful reference points for estimating height during naturalistic walking.

To investigate the role of boundaries in spatial memory for volumetric space in more detail, we analyzed how objects are replaced in test environments that have undergone geometric deformations. For instance, if the cubic baseline environment was stretched along one dimension, it is reasonable to expect a greater dispersion of responses along the same dimension, but not along the orthogonal (unchanged) one, potentially amplifying or reducing baseline anisotropies. The mode of locomotion could further influence changes to isotropy due to differences in environmental boundary processing between flying and walking. We computed anisotropy indices as the log-ratio between the vertical and horizontal MAD for all deformed environments and calculated difference scores with respect to the baseline environment (deformation ratio–baseline ratio; Fig. 2 *H* and *J*). We then tested whether the anisotropy indices changed in the expected direction and according to the scaling of the deformed environment (1.33 for stretch, 0.67 for compression). Participants’ response dispersion, indeed, changed in a dimension-specific manner approximating the scaling of the deformed environments. There were no significant differences to the expected values, except for the vertical compression condition when participants were walking (1-sample *t* test against scaling-adjusted difference to baseline: *t*_19_ = 3.76, *P* = 0.011, *d* = 0.840, all other tests: *P* > 0.238; Bonferroni-corrected for 8 comparisons). Here, the dispersion increased rather than decreased as expected from the compression, indicating that vertical compression heightened participants’ uncertainty, likely because a ground-based encoding strategy became less effective. For instance, if a target location’s height was initially far above the head and encoded relative to the ground in the baseline environment, vertical compression of the environment would force participants to adopt a novel reference strategy.

### Flying Participants Employ a Boundary-proximity Strategy Across Horizontal and Vertical Deformations.

**Fig. 3.**
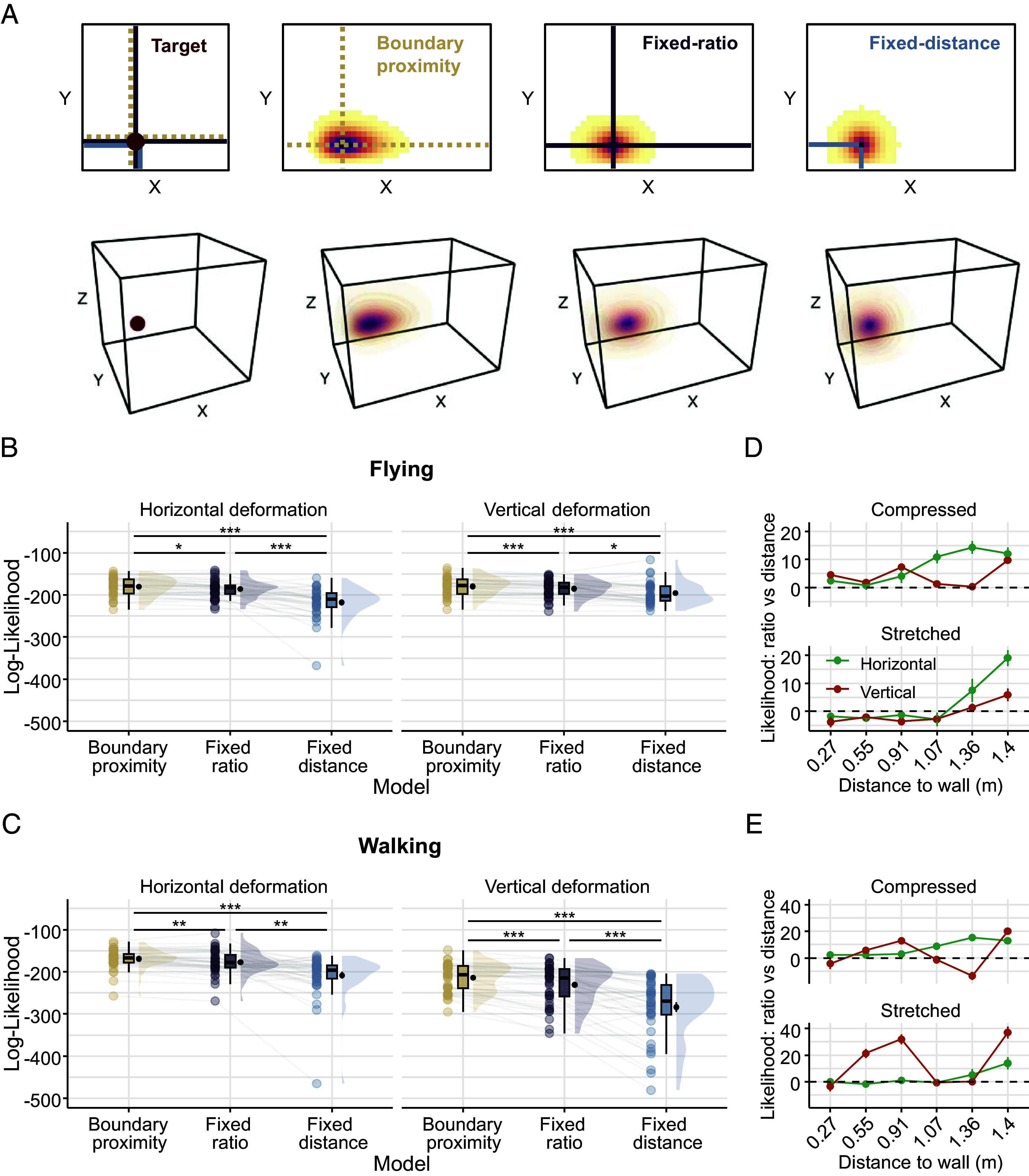
Fitting geometric models to participants’ 3D object replacements. (*A*) Three geometric models applied to the object replacement data: Fixed distance model (preserves the fixed distance of a memorized location to the nearest two walls), fixed ratio model (preserves aspect ratios between opposing walls), boundary-proximity model (preserves the proximity—inverse of nonlinear distance—to each of the walls). Heat maps represent the probability of an object replacement for a given target location from each model prediction (*Left*, red dot). *Top*: 2D projection of the probability matrix for the x- (stretched) and y-axis (nonstretched). *Bottom*: 3D-extended prediction volumes. (*B* and *C*) The boundary-proximity model shows the best fit to the replacement data across conditions. Displayed are log-likelihood model fits for each model, separately for horizontal and vertical deformations and for the flying (*B*) and walking (*C*) condition. Violin plots depict the density distribution, boxplots the median and quartiles, black dots with error bars the means ± SEM, and colored dots individual data points per condition. *P < 0.05, **P < 0.01, ***P < 0.001 (Bonferroni-corrected for multiple comparisons). (*D* and *E*) Replicating previous findings in 2D space (7), we observe a relationship between 3D distance of a target location to the walls (x-axis) and the relative fit (log-likelihood, y-axis) to the fixed ratio vs fixed distance model for different deformation types. This is especially the case for horizontal compared to vertical deformations (color-coded). The vertical stretch deformation in the walking group deviates from this pattern.

Manipulating the size and shape of a familiar task environment has been previously conducted in 2D environments to investigate the causal impact of environmental geometry on cognitive maps across species (3, 7–9, 12, 13, 36). To evaluate how object-locations are encoded in volumetric space, we extended a set of geometric models (7) from 2D to 3D space and tested their predictions on object replacements in the deformed environments (Fig. 3*A*). Each model assumes a different encoding mechanism of the object-location in the familiar baseline environment and predicts object replacements in deformed test environments that preserve their respective encoding mechanism. While the fixed distance and fixed ratio models preserve either the distances to the nearest walls or the aspect ratios to opposing walls, respectively, the boundary-proximity model encodes the proximity to all surrounding walls in a nonlinear fashion. Specifically, the model assumes that locations close to boundaries are encoded more by their fixed distance and locations in the center more by their fixed ratio (see Methods: *Modeling*). Consequently, the boundary-proximity model mimics place predictions similar to those derived from the population activity of boundary vector cells (BVCs) each tuned to a preferred allocentric direction and distance to walls (16, 18). We first fitted the model parameters to the object replacements of the baseline environment to account for baseline differences in precision to the three spatial dimensions, and then computed the model-likelihood of replacements in the deformed environments. Across all participants, the object replacements were best explained by the boundary-proximity model (one-way repeated-measures ANOVA: *F*_1.18,89.96_ = 108.963, *P* < 0.001, $\eta_{p}^{2}$ = 0.589; post hoc paired *t* test, Bonferroni corrected for 3 comparisons: proximity vs. ratio: *t*_76_ = 7.71, *P* < 0.001, *d* = 0.879; proximity vs. distance: *t*_76_ = 11.8, *P* < 0.001, *d* = 1.35; ratio vs. distance: *t*_76_ = 9.15, *P* < 0.001, *d* = 1.04). We further found a significant three-way interaction between the model, mode of locomotion, and the dimension of deformation (three-way mixed ANOVA: *F*_1.16,87.33_ = 14.459, *P* < 0.001, $\eta_{p}^{2}$ = 0.162). Post hoc paired *t* tests confirmed the superiority of the boundary-proximity model (proximity model against other models: all *P* < 0.012, Bonferroni-corrected for 12 comparisons; see Fig. 3*B*), suggesting the involvement of BVC-like computations in spatial memory during 3D deformations of the environment. Additionally, simulations based on individually fitted boundary-proximity models successfully reproduced several key effects of target-to-boundary distance and environmental scaling on response dispersion observed empirically (compare Fig. 2 and *SI Appendix*, Fig. S6; see also *SI Appendix*, Fig. S7). Similar to previous findings in 2D environments (7), the relative fit of the fixed ratio model vs. the fixed distance model was higher for the targets that were farther away from the wall in all conditions, except for the walking group in the vertically stretched environment (Fig. 3 *D* and *E*). In general, responses to vertical deformations revealed a lower fit to boundary-proximity model predictions in the walking, but not in the flying group (2-way mixed ANOVA: *F*_1,75_ = 46.088, *P* < 0.001, $\eta_{p}^{2}$ = 0.381; horizontal vs vertical for walking: *t*_39_ = 7.15, *P* < 0.001, *d* = 1.13; flying: *t*_36_ = −0.337, *P* = 1, *d = −*0.055; paired *t* test, Bonferroni-corrected for 2 comparisons).

### Walking Participants Rely on a Ground-Based Strategy in Vertical Deformations.

**Fig. 4.**
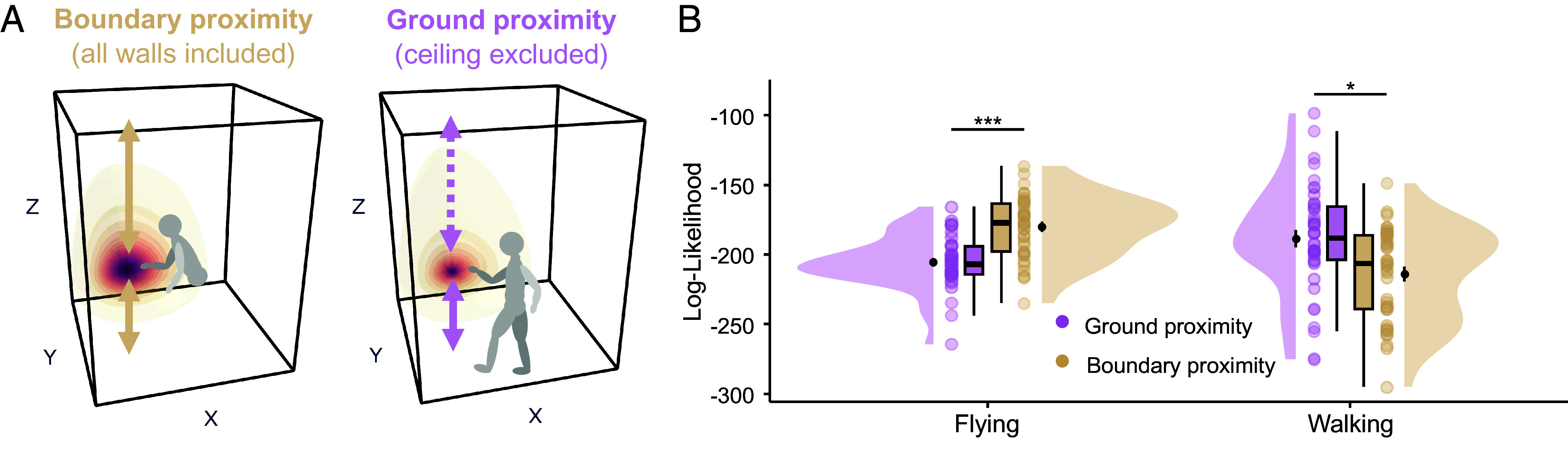
Locomotion-dependent use of vertical boundaries. (*A*) Schematic of a boundary-proximity vs ground-proximity prediction. Unlike flying participants (*Left*), walking participants (*Right*) are grounded and upright, leading to a stable relationship between their body and the ground. Thus, the usage of body-based cues could lead to an increased weighting of the ground compared to the ceiling. We modified the boundary-proximity model (yellow arrows and prediction field, *Left*) by removing the reference to the ceiling and termed it ground-proximity model. (*B*) Model comparison between the ground-proximity model and the standard boundary-proximity model in each locomotion group. The ground-proximity model showed a superior fit (high log-likelihood) for the walking group, but not for the flying group. Boxplots depict the median and quartiles, black dots with error bars the means ± SEM, and colored dots individual data points per condition. *P < 0.05, ***P < 0.001 (Bonferroni-corrected for two comparisons).

Consistent with our findings in the baseline environment, the explanatory power of the geometric models decreased for vertical deformations in the walking participants. Contrary to the flying group, walking participants maintained an upright posture aligned with the gravitational axis, allowing them to estimate the height of objects from the ground by using their body as a reference. If this is true, vertical coordinates of objects should be encoded with greater reference to the ground than to the ceiling (cf., Fig. 2*A*). Accordingly, we tested a modified version of the boundary-proximity model that preserves the proximity to all walls except the ceiling (ground-proximity model), thereby amplifying the weight of the ground’s influence (Fig. 4*A*). Notably, because body parts maintain stable vertical offsets from the ground, the ground-proximity model functions as a proxy for multiple ground-anchored body-based cues. Our analysis revealed a significant interaction between the model type and locomotion mode for the vertical deformations (two-way mixed ANOVA: *F*_1,75_ = 28.876, *P* < 0.001, $\eta_{p}^{2}$ = 0.278; Fig. 4*B*). The ground-proximity model provided a better fit for data from walking participants compared to the standard boundary-proximity model (paired *t* test: *t*_39_ = 2.86, *P* = 0.014, *d* = 0.452; Bonferroni-corrected for 2 comparisons). In contrast, data from flying participants were better explained by the standard boundary-proximity model (*t*_36_ = −11.0, *P* < 0.001, *d* = −1.81).

## Discussion

The present study employed immersive Virtual Reality to investigate the mechanisms underlying the representation of volumetric space, focusing on how different modes of locomotion (flying vs walking) and environmental geometry (cubic vs. horizontally and vertically deformed environments) affect spatial memory.

The significance of environmental boundaries for spatial memory has been well established on a 2D horizontal plane. Previous research showed that humans recall locations near boundaries with greater precision than those farther away (7, 19), which has been linked to the firing properties of boundary vector cells (BVCs) (16, 18) initially found in the rat subiculum (14). The firing of BVCs is tuned to preferred distances and directions to environmental boundaries with receptive fields that broaden as the distance from these boundaries increases, indicating diminished encoding precision. Our study suggests a similar mechanism for the encoding of volumetric space when participants were flying virtually. In the baseline environment, we observed that spatial memory precision peaked when target locations were close to any boundary—be it the ceiling, floor, or side walls. Moreover, when the test environment was vertically or horizontally deformed, flying participants adjusted their spatial memory in ways predicted by a 3D-extended boundary-proximity model which was inspired by putative 3D BVCs tuned to both vertical and horizontal boundaries. These findings imply that humans use boundaries to represent 2D and 3D space in a qualitatively similar manner when movement constraints are minimal—much like birds or fish exploring their natural habitats.

However, our data suggest that spatial encoding is not isotropic in volumetric space. Flying participants showed a lower encoding precision for the vertical compared to the horizontal dimension, contrasting with findings in flying bats, which demonstrate isotropic place fields (23). This anisotropy could stem from the spatial ecology during ontogeny and evolution of surface-dwelling animals, including biases in sensory processing, energy costs of moving against gravity, and the availability of spatial cues (33). Specifically, visual perception may be less precise when the head is tilted up or down compared to when it is oriented straight ahead (24, 25). Furthermore, despite sufficient familiarization and training, the method of virtual flying might have felt inherently unnatural for surface-dwelling humans. While upper body movements were possible, participants remained physically seated on a chair, virtually translating through optic flow but lacking the proprioceptive input associated with terrestrial walking. This flight simulation effectively breaks the stable relationship between the body and ground, making the employment of a body-axis-related strategy not useful, but potentially induced a mismatch between visual and proprioceptive feedback. Such a sensory conflict could result in heterogeneous or inconsistent responses in spatially tuned cells (37) and may disrupt spatial processing, especially in the vertical axis, where physical gravity imposes a constant influence. This raises the intriguing question of whether anisotropy would persist in environments with near-zero gravity, such as on space stations (38). In these settings, anisotropy might be undetectable due to the equivalence of all spatial axes, or it might emerge individually, depending on the subjective anchoring of “up” and “down” as reported by astronauts (34).

Anisotropy in volumetric spatial encoding may also be reflected at the neural level. One possibility is that BVCs can have preferred directions to any boundary but have larger tuning widths for vertical boundaries compared to horizontal ones. Alternatively, there may be differing numbers of BVCs tuned specifically to vertical or horizontal boundaries, akin to the distribution of head direction cells in bats, where cells tuned to horizontal directions are more abundant than those tuned to vertical ones (39). Supporting this, human fMRI research identified distinct neural sensitivity for 3D head direction during virtual flying: the thalamus and subiculum primarily encoded horizontal (azimuth) direction, with the left thalamus showing significantly stronger horizontal encoding, while the retrosplenial cortex was significantly more sensitive to vertical (pitch) direction, correlating with better behavioral vertical judgment (40). More recently, BVCs tuned to vertical or horizontal walls were detected in goldfish swimming in a quasi-planar water tank (41). Electrophysiological recordings in flying or swimming species within bounded volumetric environments could further elucidate whether BVCs are tuned to all three axes with varying tuning widths or are selectively tuned to specific planes.

In contrast to the flying condition, boundary-dependent 3D memory exhibited significant differences when participants walked, constrained to the ground plane. Here, precision for horizontal locations remained highest near boundaries, but vertical precision peaked near the center of the environment, aligning with participant’s head positions. The head, containing critical sensory organs such as the eyes for visual and the inner ear for vestibular input, provides essential cues for vertical estimation. During naturalistic walking, humans’ upright posture and gravity-aligned body axis serve as an intrinsic “ruler” for estimating vertical locations, supplementing environmental boundaries. This reliable vertical cue could compensate for the lower vertical precision in the center of an environment. In contrast to quadrupedal animals, bipedal humans can access a broader range of vertical space. Various body parts, including the eyes, head, trunk, and limbs, can serve as useful reference frames for volumetric space, putatively integrated into body-centered coordinates within the posterior parietal cortex (42–45). The abundance of body-based information under gravity may have encouraged participants to adopt more egocentric strategies, such as perceptual matching between encoding and retrieval phases or route-based navigation, as observed in the walking group. Enhancing the degree of embodiment by incorporating a visible virtual avatar could further amplify precision for vertical locations by providing additional egocentric reference cues. Future research could investigate naturalistic walking scenarios with varying levels of avatar representation—ranging from showing hands and arms to the entire torso—to better understand the role of static body-based cues in spatial memory for vertical locations. It is important to note, however, that increased vertical precision observed in the walking group, stemming from their ability to use the body as a “ruler”, is likely strongest for targets within peripersonal space. This body-based strategy becomes less effective as targets move further away, potentially leading to vertical precision patterns similar to those seen in the flying group, who lacked such reliable, gravity-anchored body-based cues for height estimation. While our current study did not directly test the impact of targets outside peripersonal space, this would be an interesting avenue for future investigation.

The distinct reference strategy during walking necessitated modification of the boundary-based computational model when predicting spatial memory in deformed environments. Unlike the 3D boundary-proximity model, which provided the best fit to the flying group’s data, the ground-proximity model which referenced only the floor but not the ceiling—best explained the walking participants’ memory. For crawling or walking animals, posture relative to the ground is a salient cue for spatial memory. Studies have shown that rodents, humans, and birds can distinguish locations on flat versus tilted surfaces, aiding their navigation and spatial memory (46–48). Due to the influence of gravity, all body cues are ultimately referenced via stable offsets to the ground. The ground-proximity model therefore serves as a proxy for multiple potential body-based referencing strategies, such as head-height references, and represents the body-to-ground relation that emerges due to gravity. Future research should explore more comprehensive models that explicitly include body-based reference cues and their weighted integration with environmental references, similar to existing work in 2D spaces (49–51). This would account for context-dependent and dynamic interactions across cue types during naturalistic walking in volumetric space. Moreover, to better disentangle the influence of different body parts and the ground, one would need to manipulate the body-to-ground relation via body-avatar deformations or varying offsets (i.e., walking in the air) in addition to geometric deformations.

Our findings emphasize the complementary roles of environmental boundaries and the gravity-aligned body axis in representing 3D space. Cognitive maps exhibit flexibility, adapting to multiple constraining factors, including the mode of locomotion, attention, and degree of embodiment. For instance, a volumetric map may be constructed when flying, whereas a quasi-planar map may be utilized when walking (52–54). Attentional shifts between local surface cues (e.g., floor texture) and global cues (e.g., environmental boundaries extending into 3D space) likely mediate the representational format. Crucially, movement constraints affect the dimension-specific weighting of cue usage for spatial encoding. The vertical dimension is special in this regard due to gravity, leading to a higher weighting of the floor during naturalistic walking. However, it is conceivable that artificially inducing movement constraints along a single horizontal dimension (e.g., x but not y) could similarly result in an uneven weighting of horizontal walls, leading to biased spatial encoding.

In conclusion, our study demonstrates that cognitive maps of volumetric spaces share a common boundary dependency with those of two-dimensional environments. However, differences in locomotion modes introduce significant variations in the use of boundary and body-based references. As terrestrial, bipedal navigators, humans encode the axis of gravity differently from the plane of locomotion, using their bodies as “rulers” to measure distance from the ground. The finding that gravity-defying conditions within the same species result in distinct vertical representations and reference frames highlights the flexibility of the cognitive mapping system, which is adapted for navigating a complex, multidimensional world.

## Materials and Methods

### Participants.

A total of 85 young healthy adults were recruited from the internal participant database of the Max Planck Institute for Human Cognitive and Brain Sciences in Leipzig, Germany. The sample size was determined by a power calculation using G*Power version 3.1.9.6 (55). We assumed a small-to-medium effect size of *f^2^* = 0.23 (directly derived from partial η^2^ = 0.05) for the interaction effect between 4 groups (flying vs walking locomotion mode, and stretch vs compression deformation) and 2 repeated measures (horizontal and vertical deformation) tested by a repeated measures ANOVA assuming nonsphericity and a medium correlation among within-subject factors of r = 0.4. This resulted in a sample size of 17 participants per group (*N*=68) to achieve a statistical power of 80%, with an error probability of α = 0.05 (two-tailed test). Data from 14 additional participants were acquired to account for potential dropouts. 3 participants did not complete the experiment due to motion sickness or fatigue. Data from 5 participants were excluded due to poor spatial memory performance at baseline, classified as having mean spatial memory errors greater than 1.5 times the interquartile range above the upper quartile of mean errors observed in the sample. Thus, data from 77 participants (40 female, 36 male, 1 nonbinary; Age: 26.182 ± 4.468 y, 19 to 35 y) entered the analysis. All participants were right-handed, had normal (or corrected-to-normal) vision, and no current or past neurological, psychiatric, or motor disorder. They were reimbursed for their participation, following institutional procedures. To minimize the variability in spatial abilities and movement dynamics due to differences in body height, we included only participants with a height between 165 cm and 185 cm. All participants gave informed consent, and the full study protocol was approved by the Ethics Advisory Board of the Medical Faculty of the University of Leipzig. Participants were pseudorandomly assigned to 4 experimental groups of approximately equal size, with no significant differences in demographic variables and body measures between groups (all *P*’s ≥ 0.08). A complete record of group-specific sample characteristics is provided in *SI Appendix*, Table S1.

### Experimental Setup.

The experiment took place in a Virtual Reality (VR) lab at the Max Planck Institute for Human Cognitive and Brain Sciences in Leipzig, Germany (*SI Appendix*, Fig. S1*A*). Participants wore a Pico Neo 3 Pro Eye HMD (3664 × 1,920 pixels, horizontal field of view: 98°, vertical field of view: 90°, refresh rate: 90 Hz, weight: 620 g; Pico Interactive, San Francisco, CA, https://www.picoxr.com) displaying custom-made virtual environments created in Unity3D (version 2021.3.3f1; Unity Technologies, San Francisco, CA) and rendered on a stationary computer (AMD Ryzen 5 3600 6-core 3.6 to 4.2 GHz processor, NVIDIA GeForce RTX 3090 graphics card, Intel I350 network card, running Microsoft Windows 10). Within the virtual environment, the participants’ viewpoint was continuously updated based on their real-time head location and movement in virtual space, allowing for naturalistic behavior. Full-body movements were recorded at a sampling rate of 200 Hz (downsampled to 40 Hz) using a Vicon MoCap system (Vicon, Oxford, UK; https://www.vicon.com/) with 9 Vero v2.2 infrared cameras at a resolution of 2.2 MP covering a tracking volume of approximately 4 × 5 × 2.5 m. During the experiment, participants wore passive retroreflective markers attached to 13 to 14 rigid bodies, the HMD, and a handheld motion controller (*SI Appendix*, Fig. S1*B*). 3D motion data were live-streamed into the SteamVR-based application, which maintained a wireless connection to the HMD via the PCVR streaming software Pico Link and a Wi-Fi 6 router (ASUS RT-AX82U). The VR application was remotely controlled and monitored by the experimenter.

A follow-up debriefing questionnaire was created using SoSciSurvey version 3.5.01 (56) (www.soscisurvey.de) and presented on a standard computer screen (2,560 × 1,440 pixels).

### Study Design and Virtual Environments.

We implemented a 2 × 2 × 2 mixed factorial design manipulating the within-subject factor spatial dimension (horizontal vs. vertical) and the between-subject factors deformation type (stretch vs. compression) and locomotion mode (walking vs. flying). An overview of the experimental conditions is provided in Fig. 1*C*.

During training, participants learned the 3D location of multiple virtual objects within a symmetrically shaped cubic baseline environment. During the test, their spatial memory was probed both in the familiar environment and in geometrically deformed environments (see Object-location memory task in volumetric space). While for one group of participants the test environment was stretched (groups 1 and 2), for another group it was compressed (groups 3 and 4). For all participants, both the vertical and horizontal dimensions of the environment were deformed separately. The task was performed in two distinct locomotion modes (Fig. 1*D*), which differed relatively in their constraints by gravity and the resultant body-based cues. One group of participants physically walked with their bodies maintaining a stable relationship to the ground constrained by gravity (groups 1 and 3). As a natural mode of locomotion for humans, it involved translation with two horizontal degrees of freedom and primarily yaw rotation, providing rich lower-limb proprioception and rhythmic vestibular/motor feedback, with vertical displacement through postural adjustments or arm extension for targets. Another group of participants was able to fly virtually through space, mimicking the relatively unconstrained locomotion mode of winged animals, thus offering no static body-to-ground alignment cues (group 2 and 4). These participants sat in a motion-captured rotatable chair and controlled their 3D locomotion direction by rotating their head (yaw & pitch) and forward/backward translation using the controller’s joystick. Consequently, they received dynamic self-motion cues from head and upper torso movements but lacked lower-limb proprioception and rhythmic vestibular/motor feedback associated with terrestrial walking. The maximum speed of movement was constant across all environments at 1 m/s, reached after 0.5 s after the initial joystick push/pull, with a smooth acceleration at a rate of 1.5 m/s^2^. All participants could reach positions within peripersonal space by extending the arm upward or bending downward. Participants in the walking group were able to extend the virtual arm controller like a wand if a target could not be reached by physically extending the arm upward (controller position ≥15 cm above the head and forward tilt angle of ≤55°).

Spatial memory was assessed in 5 different virtual environments, one baseline cube and four deformed cuboids (horizontally stretched, horizontally compressed, vertically stretched, vertically compressed). The baseline environment consisted of a cubic enclosure (3 × 3 × 3 m) made of six equally sized walls. These walls were given unique colors and tile textures to facilitate orientation and provide optical flow during locomotion (*SI Appendix*, Fig. S1*G*). The assignment of colors and textures to walls was randomized across participants but fixed throughout the experiment. The deformed environments were exact duplicates of the baseline environment, except that they were uniformly stretched or compressed by a fixed rescaling factor of 0.33 (approximately 1 m) along either the horizontal (x) or vertical (z) axis, relative to the baseline environment. All virtual environments were symmetrically illuminated, so that light could not be used as a directional cue. No local landmarks were provided at any time to enforce spatial learning based on the geometry of the environment. Detailed characteristics of the virtual environments are shown in *SI Appendix*, Table S2.

### Experimental Protocol.

Participants completed the following steps in consecutive order: preparation, main task, and debriefing. The entire experimental session took 60 to 210 min (M = 148 min; SD = 30 min) with 42 to 162 min (M = 84 min, SD = 24 min) in VR.

#### Preparation.

Upon arrival, participants read the written task instructions and were informed about the general objectives, procedure, and potential risks (e.g., cybersickness) participating in the study. After giving consent, the experimenter recorded several body measurements (body height, arm span, inseam length, hip width, and arm length) and attached the rigid bodies to the participants’ body, with the configuration varying slightly between conditions (*SI Appendix*, Fig. S1*B*). Participants did not wear shoes throughout the experimental session. We then fitted a virtual avatar to the participant’s T-pose to scale a simple digital 3D model of the participant’s body based on individual measurements. Importantly, the avatar was not visible to the participant during the entire experiment. All participants started the experiment from the center of the lab’s horizontal plane, either standing or sitting in a chair. Throughout the session, condition-specific task instructions were delivered via a virtual display attached to the animated controller (Fig. 1*B*).

#### Object-location memory task in volumetric space.

Participants performed an object-location memory task, which we extended from 2D to 3D space. The general task structure followed (57) and can be divided into four successive phases: familiarization, initial learning, feedback, and test (Fig. 1*A*). Videos of exemplary task trials for both locomotion modes can be found on the Open Science Framework (https://osf.io/r7ubv/).

*Familiarization*. Participants were spawned into the virtual environment and practiced task-relevant interactions and movements during the familiarization phase. In each trial, they were instructed to move freely to a floating green ball and collect it by pointing the tip of the animated controller into the ball until it lights up and then pressing a button. After collection, the ball reappeared at a different location within the virtual environment. Participants completed 6 familiarization trials (duration: M = 1.8 min, SD = 1.2 min).

*Initial learning.* In each trial of the subsequent initial learning phase, participants moved to a start location, indicated by a green ball. Once collected, the ball disappeared and a target object was displayed at its predefined location. Participants were asked to move to the object, memorize its location, and collect it. There were 6 trials, one for each target location (duration: M = 3.0 min, SD = 2.4 min).

*Feedback.* Next, learning was continued through feedback-based training. On each trial of the feedback phase, participants again collected a green ball at a 3D start location. A 3D model of the object then appeared attached to the controller, prompting them to place the object at the remembered location. At the same time, the name of the object was printed on the controller’s display. Once the response was confirmed by pressing a button (“response”), its location was marked in the form of a hologram as the object moved to its correct location. In addition, accuracy-related feedback was printed on the controller’s display via one of five smileys and a numerical error value. The feedback was based on the 3D Euclidean distance between the response and the correct location, using predefined thresholds (0 to 0.17 m = very good, 0.17-m = good, 0.27-m = intermediate, 0.47 to 1 m = bad, >1.06 m = very bad). These thresholds were derived via $3D\;Euclidean\;error<\frac{d_{\max}}{{\left\{5,4,3,2,1\right\}}^{2}}$, where $d_{\max}$ represents the maximum possible distance in a 3 × 3 m plane. Finally, participants recollected the object before the next trial began. In total, there were 48 trials with 8 repetitions per target location (duration: M = 27.6 min, SD = 10.8 min).

*Test.* Ultimately, spatial memory was probed during the test phase, which served as a readout of spatial memory, i.e., the result of training. Trials in the test phase followed the same timeline as the feedback trials, except for feedback and re-encoding. Participants completed a total of 5 blocks, each consisting of 18 trials. Within each block, participants performed 6 consecutive trials in one of the three virtual environments (baseline, vertical deformation, horizontal deformation) before switching to the next environment. The order of the environments was randomized within each block. This design resulted in a total of 90 test trials across all blocks (duration: M = 43.2 min, SD = 12.6 min).

Participants learned objects at 6 fixed target locations that were freely floating in volumetric space (*SI Appendix*, Fig. S1*C*). These locations and their distances to the nearest wall were evenly distributed across spatial dimensions, with the median coordinates close to the center of the environment and no significant difference in uniformity between spatial dimensions (*SI Appendix*, Fig. S1 *D*–*E*). The minimum Euclidean distance to walls and between targets was 26 cm, resulting in a coverage of 5/8 of the space and smaller than larger intertarget distances (M = 0.747 m, SD = 0.585 m; *SI Appendix*, Fig. S1*F*). 3D models of everyday-life objects (camera, alarm clock, apple, measuring tape, wooden elephant, and teapot; *SI Appendix*, Fig. S1*H*) were taken from the Poly Haven asset library (www.polyhaven.com; published under the free license CC0). The mapping between objects and target locations was randomized across participants, and we observed differences in spatial memory error only for the target location, not for object identity (*SI Appendix*, Fig. S3 *A* and *B*). Start locations were pseudorandomly sampled so that the minimum distance between start and target location was 1 m and the entire environment was covered in each experiment phase. The order of trials was organized into sets of 6 trials, with each object-location sampled once in each set and no two consecutive trials containing the same object-locations. There was no time limit for completing trials and no virtual teleportation between trials.

#### Debriefing.

At the end of the experiment participants completed a short questionnaire on a computer screen. We asked participants about their deformation perception (whether the deformation was noticed and, if so, its perceived direction and magnitude), spatial perspective taking (egocentric vs allocentric vs mixed perspective), boundary-related strategies (fixed distance vs fixed ratio vs mixed strategy), as well as self-rated motion sickness, immersion, and motivation. Debriefing-related descriptions and results are summarized in *SI Appendix*, Fig. S9.

### Data Processing and Analysis.

Statistical analyses and visualization were performed in R 4.2.2 using the RStudio developer environment (version 2023.06.0). Unless otherwise noted, we used an alpha level of 5 % and two-sided tests. In case of multiple comparison testing, we applied the Bonferroni correction.

#### Training and test performance.

Baseline performance, i.e. spatial memory accuracy, in volumetric space was assessed by calculating the Euclidean distance in 3D space between the correct target location and the replacement locations. We first computed the error in spatial memory, averaged over target locations, for each repetition during the feedback phase. We then tested for differences across repetitions (pooled) and between walking and flying using a 1-way repeated measures ANOVA and a two-sample *t* test, respectively. Successful encoding, resulting from training, was assessed by comparing the pooled spatial memory errors in the baseline test environment against the mean chance level via a 1-sample *t* test. The chance level was determined by simulating 10,000 random responses for each target location and calculating the corresponding Euclidean error.

#### Locomotion- and geometry-dependent anisotropy.

To compare the precision of the horizontal and vertical encoding in volumetric space, we computed the median absolute deviation (MAD) for each target location within participants. This dispersion measure is robust to outliers, and does not require a reference to the correct value, making it suitable for deformed testing environments where responses cannot be compared to correct locations. The MAD reflects the consistency of spatial memory (analogous to the precision, or width of receptive fields of spatially tuned cells). We compared the MAD for the z-axis (vertical dimension) to the average of the x and y axes (horizontal dimension) in two locomotion modes using a two-way mixed ANOVA followed by pairwise post hoc *t* test.

Next, we evaluated whether the MAD is dependent on the distance between a target location and the environmental boundaries and whether this relationship differs across vertical and horizontal dimensions as well as locomotion modes. Separately for the horizontal and the vertical dimension, and each participant, the distance of each object to the closest boundary was computed and we correlated these distance values with the MAD values. The individual Spearman correlation values were averaged for each locomotion group and tested against zero in a 1-sample *t* test. Similarly, we tested for a relationship between the vertical MAD and head-to-target distance. Here, we computed the vertical distance between each target and participant’s height (top of the head, measured prior to the experiment; see *SI Appendix*, Table S1).

Subsequently, we tested how response dispersion changes with deformations of the environment, that is, whether the change in MAD between baseline and the deformed environments is proportional to the actual deformation factor (i.e., 33%). To this end, we computed anisotropy indices (AI) for each participant and environment by taking the log-ratio of the MAD between the vertical and horizontal dimensions (AI = ln(MAD_Vertical_) − ln(MAD_Horizontal_). Positive AI values indicate a higher relative vertical-to-horizontal dispersion (which is the case for a vertical stretch or a horizontal compression), whereas negative values indicate a lower relative vertical-to-horizontal dispersion (vertical compression or horizontal stretch). A value of zero would indicate isotropy. We then computed the deformation-induced AI change by subtracting the baseline AI. These ΔAI values were compared, via Bonferroni-corrected *t* tests within each locomotion group, to the four expected ΔAI values arising from a 33% stretch or compression along one axis (VS = +0.285, HC = +0.400, HS = −0.285, VC = −0.400).

#### Navigational parameters.

*State occupancy*. We divided the environment into 50 × 50 × 50 equally sized bins and calculated the number of visited bins in the baseline testing environment across trials and participants. Location visits were based on the position of a collector rigid body, since it is most comparable between both locomotion conditions.

*Path length*. The total path length between the starting location and replacement location in the baseline testing environment was computed as cumulative frame-wise 3D Euclidean distance and averaged for each participant.

*Path dissimilarity*. For each target location we computed the dissimilarity of movement paths between the starting and replacement location across trials via dynamic time warping [DTW, via the *dtw* function in *R,* (58)]. DTW computes the similarity between two temporal sequences that may vary in speed or timing. It optimally aligns the sequences by stretching or compressing segments based on a warping function, thereby minimizing the cumulative distance between aligned points. First, the time series was downsampled from 40 Hz to 4 Hz. Then, we computed the DTW dissimilarity for each pair of trials for a particular target location before averaging the values for each participant.

*Heading difference*. We computed the circular average horizontal (azimuth) and vertical (elevation) orientation of the head at object placement during the feedback learning and test phase in the baseline environment and calculated its angular difference.

We tested for differences between the two locomotion modes in these parameters via two-sample *t* tests. To evaluate whether individual differences in navigational parameters are related to the processing of the vertical axis, we computed Spearman’s *Rho* correlation values between the vertical MAD of responses and each navigational parameter.

#### Modeling.

Participants can remember the location of objects using different geometric relations or features such as the absolute distance to the closest walls or the ratio of the distances to opposing walls. We tested which of several geometrical models best explains the object replacement data in the deformed environment (7). In a deformed environment, locations that share features with the encoding environment are more likely to be chosen for object placement whereas locations with low feature similarity to the encoding environment are less likely to be chosen. For each location in the encoding environment, we can generate a prediction field with the shape of the testing environment that has this gradual likelihood. This was done for the following models:

*Fixed distance.* This model represents locations as feature vectors that contain a location’s perpendicular distance *d* to the closest wall on each environmental axis (CG: Ceiling–Ground, NS: North–South, EW: East–West). In a 3D environment, this vector is of length 3:

$$V=\left[d_{\mathrm{CG}},d_{\mathrm{NS}},d_{\mathrm{EW}}\right].$$

*Fixed ratio.* Locations are represented as three-dimensional vectors that encode the ratio of distances *r* to opposing walls on each environmental axis (e.g., *r*_NS_ as the proportion of the object’s distance to the north wall to the distance between the north and south wall).

$$V=\left[r_{\mathrm{CG}},r_{\mathrm{NS}},r_{\mathrm{EW}}\right].$$

*Boundary-proximity.* The boundary-proximity model developed by Hartley et al. (7) produces prediction fields similar to those derived from a population-model of boundary-vector cells (16, 18). Locations are encoded by their proximity to all walls (ceiling, ground, north, south, east, west). Proximity is defined as 1/(*d* + *c*), with the distance *d* to a wall and the constant *c*. In a three-dimensional environment with six walls each location is represented by a six-dimensional vector:

$$V=\left[\frac{1}{d_{C}+c},\frac{1}{d_{G}+c},\frac{1}{d_{N}+c},\frac{1}{d_{S}+c},\frac{1}{d_{E}+c},\frac{1}{d_{W}+c}\right].$$

The boundary-proximity model behaves like a fixed distance model for objects close to the walls and like a fixed ratio model for objects at the center [see (7) and Fig. 3 *D*–*E*]. The *c* parameter controls the degree to which predictions align more closely with fixed distance or fixed ratio predictions. To remain consistent with the approach of ref. 7, we did not fit the constant *c* to our data. Instead, like ref. 7, we fixed its value at the half of the cubic baseline environment’s axis size (1.5 m), which provides a compromise between predictions based on fixed distances and those based on fixed ratios.

*Ground-proximity* (*ceiling-excluded*). This model is a modification of the boundary-proximity model which reflects the direction of gravity in the 3D environment. It tests the assumption that, because the body is stably anchored to the ground, walking participants may benefit from more precise object-to-ground estimation via body cues. The ground hereby serves as a proxy for multiple body-based references (e.g., head or limb height) that are ground-anchored and maintain stable offsets within a participant. In contrast to other potential references (e.g., environmental midpoint height), the ground provides a universal zero-point and is the essential surface on which terrestrial animals locomote. Accordingly, compared to the original boundary-proximity model, we retained the distance to the ground and excluded the distance to the ceiling, resulting in a five-dimensional vector for each object-location:

$$V=\left[\frac{1}{d_{G}+c},\frac{1}{d_{N}+c},\frac{1}{d_{S}+c},\frac{1}{d_{E}+c},\frac{1}{d_{W}+c}\right].$$

*Model fit and comparison.* We generated a prediction field (Fig. 3*A* and *SI Appendix*, Fig. S5) for each model to compute the likelihood of an object replacement in a given testing environment. First, the encoding environment was down-sampled to a 25 × 25 binned grid and the deformed environments accordingly (e.g., 33 × 25 binned grid in a horizontally stretched environment). Then, we computed the model-specific feature vectors for each location in the encoding (cube) as well as the deformed environments (rectangular cuboids). The *L^2^* norm between two vectors, one from the encoding and one from the testing environment, was computed and the repetition of this process for all cross-environment combinations yielded a representational dissimilarity matrix *D*_xyz_. This matrix was then converted into a similarity matrix by

$$S_{\mathrm{xyz}}=\max\left(D_{\mathrm{xyz}}\right)-D_{\mathrm{xyz}}.$$

*S*_xyz_ was turned into a probability matrix *P*_xzy_ by applying a softmax function:

$$P_{\mathrm{xyz}}=\frac{\exp\left(\frac{S_{\mathrm{xyz}}}{T_{x}}\right)}{\sum_{x\prime }\exp\left(\frac{S_{x\prime yz}}{T_{x}}\right)}\cdot \frac{\exp\left(\frac{S_{\mathrm{xyz}}}{T_{y}}\right)}{\sum_{y\prime }\exp\left(\frac{S_{xy\prime z}}{T_{y}}\right)}\cdot \frac{\exp\left(\frac{S_{\mathrm{xyz}}}{T_{z}}\right)}{\sum_{z\prime }\exp\left(\frac{S_{xyz\prime }}{T_{z}}\right)},$$

where *T* are the dimension-specific temperature parameters that control the sharpness of the probability distribution (low values approximating peak predictions and high values uniformity). The three temperature parameters were first fit to the object replacements in the baseline test environment, for each model and participant separately, using the Limited-memory BFGS algorithm (59) via the minimize function of the *scipy* Python package.

The fitted models were subsequently applied to the deformed environments to receive a log-likelihood (LL) estimate for each participant and model. The LL estimates were compared between the models in repeated-measures ANOVAs and subsequent post hoc *t* test (with Bonferroni-based family-wise error correction). Furthermore, to confirm that the winning model could reproduce key behavioral patterns, we simulated trial-level responses from individually fitted boundary-proximity model parameters. For each empirical trial, we drew 100 responses out of the model-based probability matrix of the respective environment. We then analyzed these synthetic datasets in the same way as the empirical data (see *SI Appendix*, Figs. S6 and S7 for simulations based on the ground-proximity model). To assess absolute model performance, we additionally compared each model’s LL to that of a chance-level baseline model, defined as a uniform probability distribution over all available spatial bins. From these comparisons, we derived a pseudo-R^2^ measure (Pseudo-R^2^ = 1 − LL_model_/LL_chance_) that quantifies how well each model explained the observed responses relative to this uniform baseline (with pseudo-R^2^ values approaching 1 indicating perfect model prediction, values around 0 indicating chance-level performance, and negative values indicating worse-than-chance performance). Statistical significance of pseudo-R^2^ values greater than zero was assessed using one-sample *t* tests, with Bonferroni corrections for multiple comparisons (see *SI Appendix*, Fig. S8 for results). The modeling analyses and visualizations were run by custom Python (3.9) scripts.

## Supplementary Material

Appendix 01 (PDF)

## Data Availability

Anonymized behavioral data and code are available at the Open Science Framework (OSF): https://doi.org/10.17605/OSF.IO/R7UBV (60).
