# Supplementary material for: Locomotion-dependent use of geometric and body cues in humans mapping 3D space
Source: Proc Natl Acad Sci U S A. 2025 Dec 19;122(51):e2505613122. doi: 10.1073/pnas.2505613122 (PMC12745749; doi:10.1073/pnas.2505613122)
Supplement: Supplementary file 1 — Appendix 01 (PDF) [file pnas.2505613122.sapp.pdf]

Locomotion-dependent use of geometric and body cues in humans mapping 3D space

Volker Reisner[1]*, Theo AJ Schäfer[1,2]*, Leonard König[1], Misun Kim[1,3+] & Christian F Doeller[1,4+]

[1] Max Planck Institute for Human Cognitive and Brain Sciences, Leipzig, Germany

[2] Institute of Psychology, University of Hamburg, Hamburg, Germany

[3] Institute of Cognitive Neuroscience, University College London, London, United Kingdom

[4] Kavli Institute for Systems Neuroscience, Centre for Neural Computation, The Egil and Pauline Braathen and Fred Kavli Centre for Cortical Microcircuits, Jebsen Centre for Alzheimer's Disease, Norwegian University of Science and Technology, Trondheim, Norway

* Contributed equally

+ Jointly supervised the work

Correspondence:

reisner@cbs.mpg.de, tschaefer@cbs.mpg.de, misun.kim.13@ucl.ac.uk, doeller@mpg.cbs.de

Supporting Information

Supplementary Figure 1

Supplementary Figure 2

Supplementary Figure 3

Supplementary Figure 4

Supplementary Figure 5

Supplementary Figure 6

Supplementary Figure 7

Supplementary Figure 8

Supplementary Figure 9

Supplementary Table 1

Supplementary Table 2

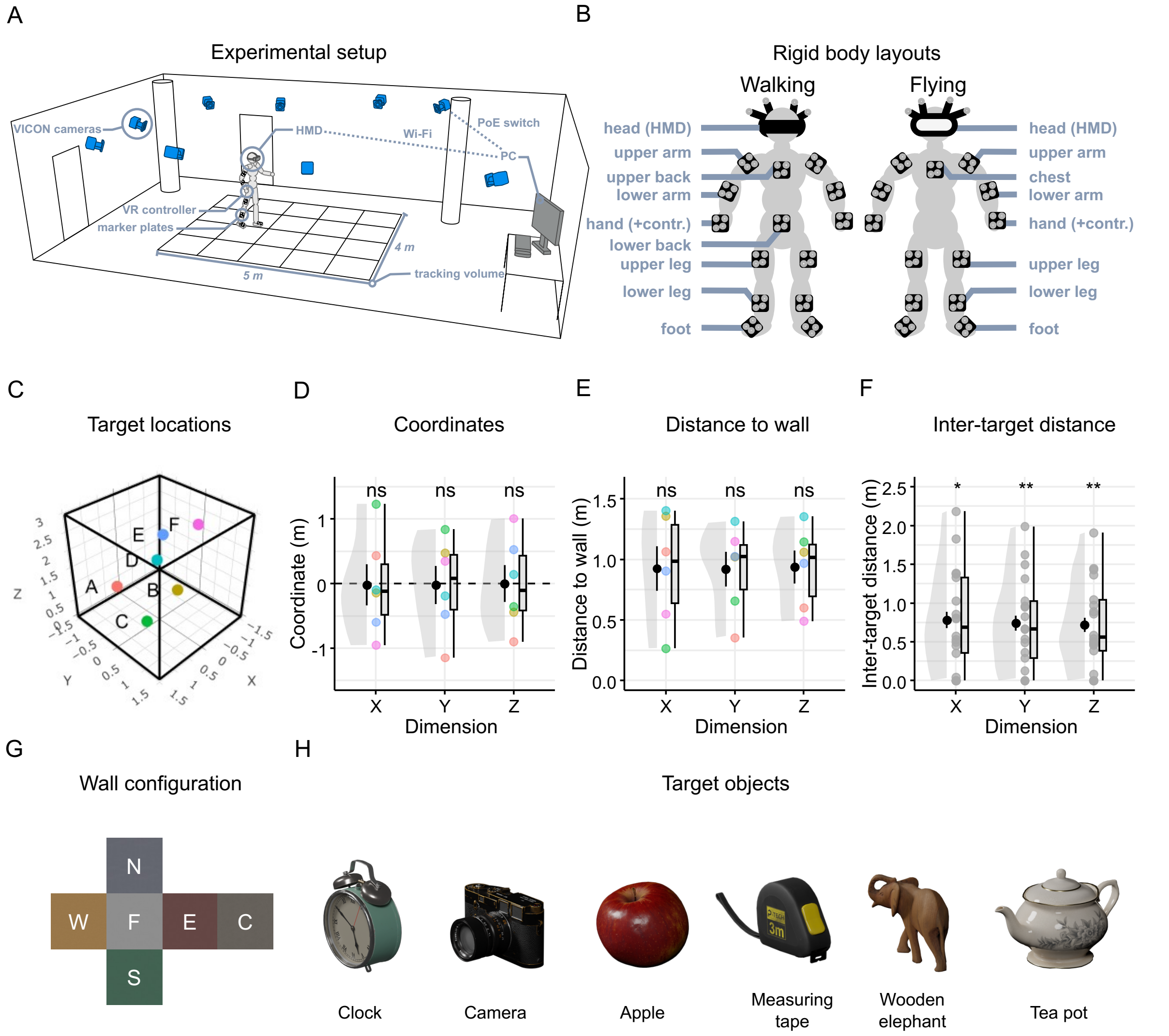

Figure S1. Experimental setup and predefined object-locations. A: Schematic of the Motion-Capture (MoCap)-featured VR lab and used equipment. **B:** Rigid body layouts for participants of the flying (13 rigid bodies) and walking (14 rigid bodies) groups, illustrating the placement of markers on different parts of the body. Note that the flying participants did not wear a marker on their lower back because they were sitting in a rotating chair whose position we tracked instead (not shown here). **C:** Fixed set of six target locations (colored and labeled dots) within the cubic baseline environment. **D-F:** We assessed whether the target-related values (coordinates, distance-to-wall, inter-target distance) within each dimension were uniformly distributed using the Kolmogorov-Smirnov (KS) test. To compare uniformity across the dimensions, we performed pairwise permutation tests. For each comparison, we calculated the absolute difference between the KS statistics of the two dimensions. We then generated 1,000 permuted datasets by randomly shuffling the distances between dimensions and recalculated the KS statistic differences. **D:** Coordinate distribution of target locations (colored dots) for each spatial dimension. Coordinates of any dimension did not significantly deviate from uniformity (*KS*-test for uniformity; X-dimension: D = .226, P_{adj} = 1, Bonferroni-corrected for 3 comparisons; Y-dimension: D = .167, P_{adj} = 1; Z-dimension: D = .168, P_{adj} = 1) with no differences in the magnitude of uniformity across dimensions (Permutation-based comparison between pairs of dimensions: Mean ΔD = .04 m, all P_{adj}'s = 1, Bonferroni-corrected for 3 comparisons). **E:** Distribution of distances between targets (colored dots) and nearest wall for each spatial dimension. Distances of any dimension did not significantly deviate from uniformity (X-dimension: D = .293, P_{adj} = 1, Bonferroni-corrected for 3 comparisons; Y-dimension: D = .333, P_{adj} = 1; Z-dimension: D = .284, P_{adj} = 1) with no differences in the magnitude of uniformity across dimensions (Mean ΔD = .096 m, all P_{adj}'s < .744, Bonferroni-corrected for 3 comparisons). **F.** Distribution of distances between target locations for each spatial dimension. Distances were non-uniformly distributed (X-dimension: D = .276, P_{adj} = .025, Bonferroni-corrected for 3 comparisons; Y-

dimension: D = .308, P_{adj} < .01; Z-dimension: D = .3, P_{adj} < .01) with more small than large inter-target locations but no differences between dimensions (Mean ΔD = .017 m, all P_{adj}'s = 1, Bonferroni-corrected for 3 comparisons). Violin plots depict the density distribution, boxplots the median and quartiles, black dots with error bars the means ± SEM, and colored dots individual data points per condition. * P < .05, ** P < .01. **G:** Example baseline wall configuration; F = Floor, C = Ceiling, N = North, S = South, E = East, W = West. **H:** 3D models of target objects used in the experiment.

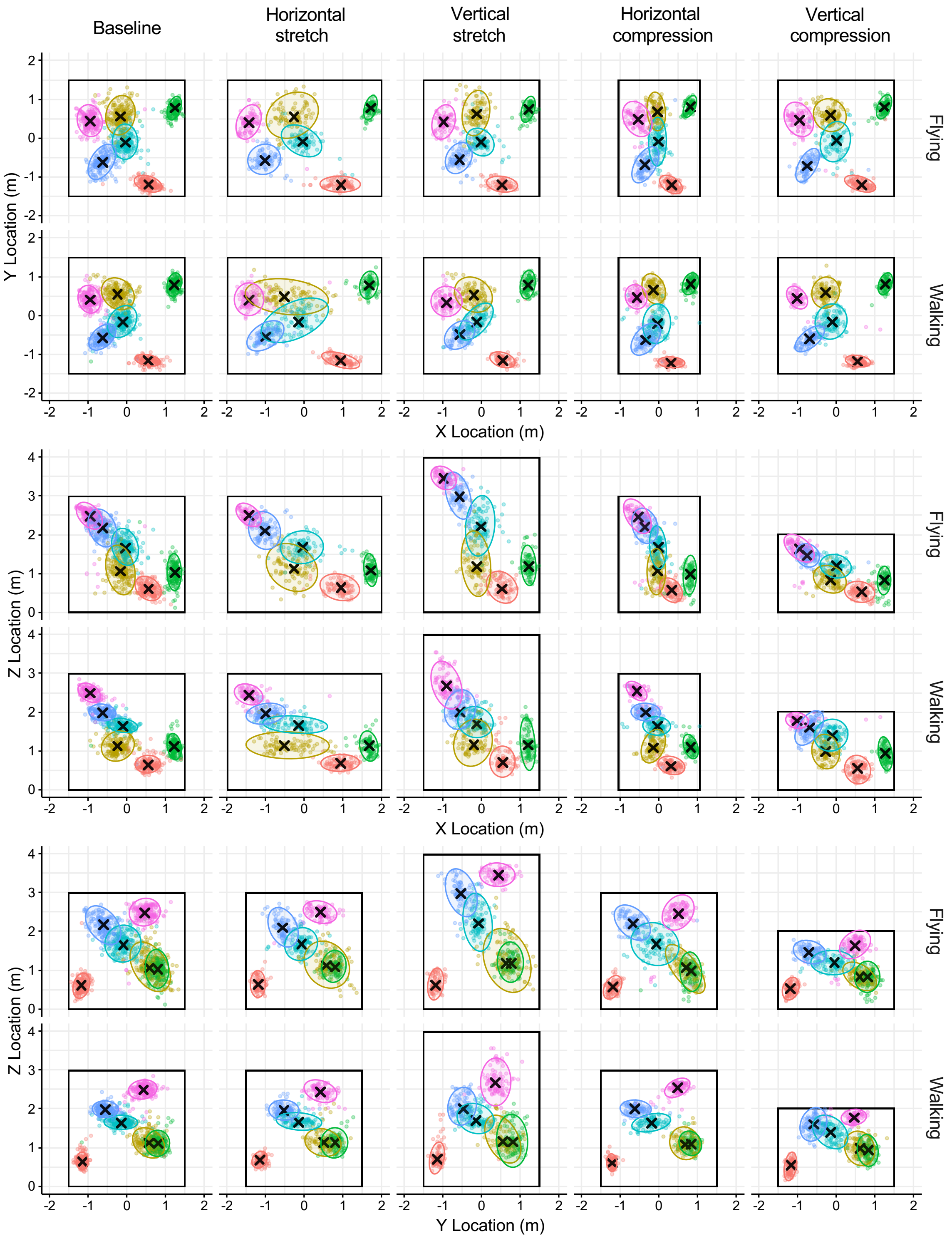

Figure S2. 2D projections of object replacements at test. Object replacements (colored dots) in the baseline environment (first column) and deformed environments (columns 2-5) of the flying and walking groups (first and second row of each subplot, respectively). Each dot represents one participant's single response, with 5 replacements for each target location. Colored ellipsoids based on multivariate *t*-distributions, each covering 95% of the data, were fitted to each target's median response (superimposed crosses). Data is separately plotted for each 2D projected view: Top view (XY; row 1-2), front view (XZ; row 3-4), side view (YZ; row 5-6).

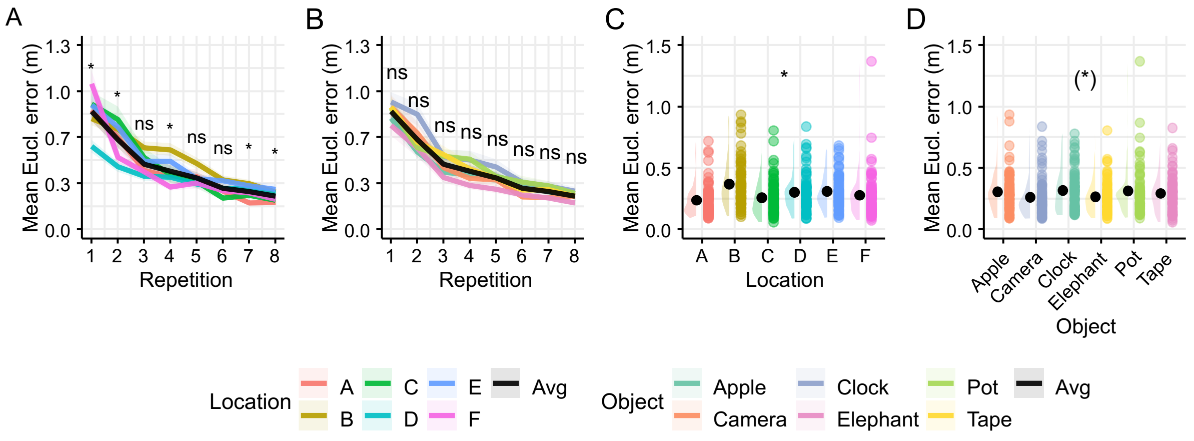

Figure S3. Location- and object-specific spatial memory performance at baseline. A: Training accuracy (mean Euclidean error) of each target location as a function of repetition. Errors significantly differed between target locations at all repetitions, except for 3, 5 and 6 (1-way repeated measures ANOVAs: all *P*'s < .05, Bonferroni-corrected for 8 comparisons). **B:** Training accuracy (mean Euclidean error) of each object (e.g. apple, clock) as a function of repetition. Errors did not differ between objects at all repetitions (1-way repeated measures ANOVAs: all *P*'s > .10, Bonferroni-corrected for 8 comparisons). **C:** Test accuracy for each target location. Errors were significantly different across target locations (1-way repeated measures ANOVA: $F_{4.31,327.23}$ = 10.356, P <.001, η_p^2 = .12). **D:** Test accuracy for each object. Although errors differed weakly between objects ($F_{4.37,332.47}$ = 2.544, P = .04, η_p^2 = .032), none of the post-hoc pairwise comparisons survived Bonferroni-correction for multiple comparisons (all *P*'s > .2). This, along with the small effect size, suggests that differences in errors across objects are not statistically robust. Color-coded lines depict means ± SEM. Violin plots depict the density distribution, black dots with error bars the means ± SEM, and colored dots individual data points per condition. * P < .05.

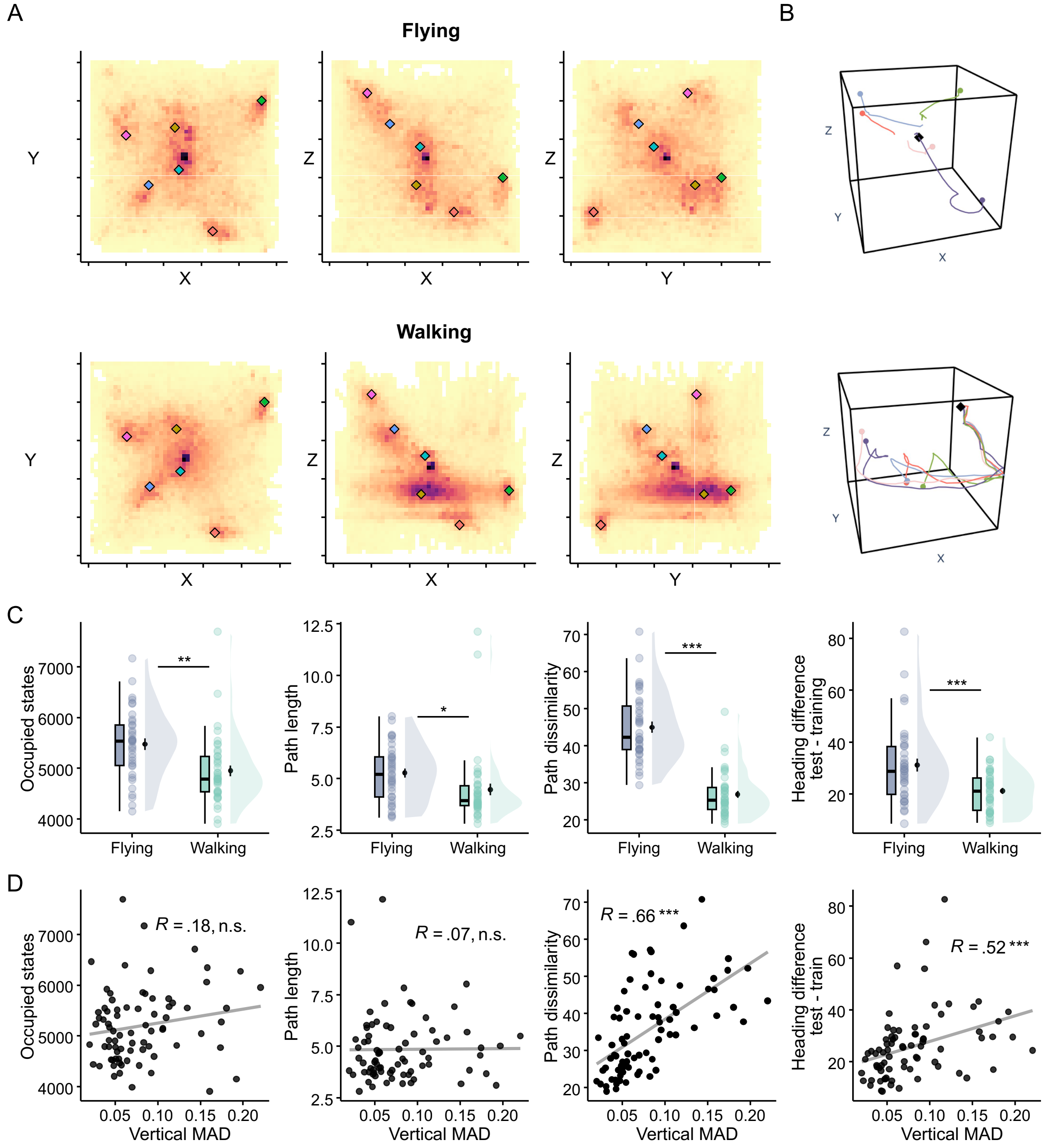

Figure S4. Navigation behavior differs between locomotion modes. A: Location-occupancy of the collector in the baseline environment (downsampled to 50 bins), normalized within and summed across participants and projected onto 2D planes, with darker shades reflecting more location visits. Colored diamonds depict the target locations. **B:** Example trajectories of two participants (top: flying; bottom: walking) in the baseline environment engaging distinct navigational strategies. Colors refer to different trials, circles to the starting location and the black diamond to the target location. **C:** Locomotion-dependent differences in state occupancy (left; $t_{74.5}$ = 3.38, P = .001, d = .771), path length (center-left; $t_{71.8}$ = -2.24, P = .028, d = -.508), path dissimilarity (center-right; $t_{61.7}$ = 10.1, P < .001, d = 2.32) and test vs. training heading difference (right; $t_{52.4}$ = 3.57, P < .001, d = .823). **D:** Correlations (Spearman's Rho) between navigational variables from C with vertical median absolute deviation (State occupancy: Spearman's *Rho* = .18, S = 62696, P = .126; Path length: Spearman's *Rho* = .07, S = 70874, P = .554; Path dissimilarity: Spearman's *Rho* = .66, S = 25672, P < .001; Heading difference: Spearman's *Rho* = .52, S = 36594, P < .001; all other P > .126). * P < .05, ** P < .01, *** P < .001.

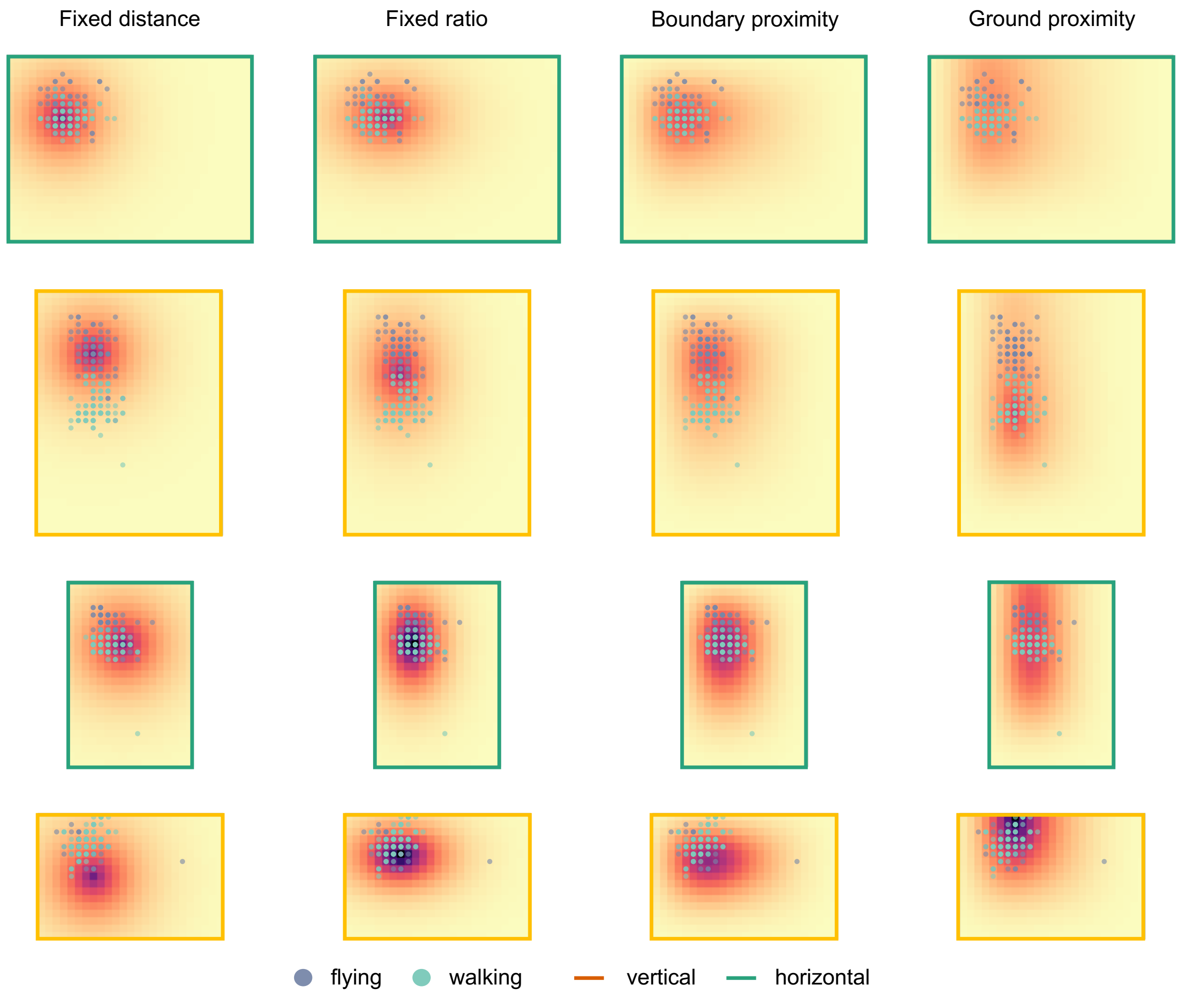

Figure S5. Model prediction fields. Heatmaps represent the (unfitted) prediction fields derived from various geometric models (column 1: *Fixed distance*, 2: *Fixed ratio*, 3: *Boundary-proximity*, 4: *Ground-proximity*) under different environmental deformations (rows 1-2: stretch, 3-4: compression) for one example target location (see Fig. 1E, object E). Darker colors indicate areas of higher replacement probability. Superimposed dots depict binned object replacements of walking (cyan) and flying (grayish blue) participants. The color of the frames reflects the type of environmental deformation: vertical (orange) versus horizontal (green). Prediction fields of each model were fitted to object replacements of walking and flying participants (see Methods section under "Modeling").

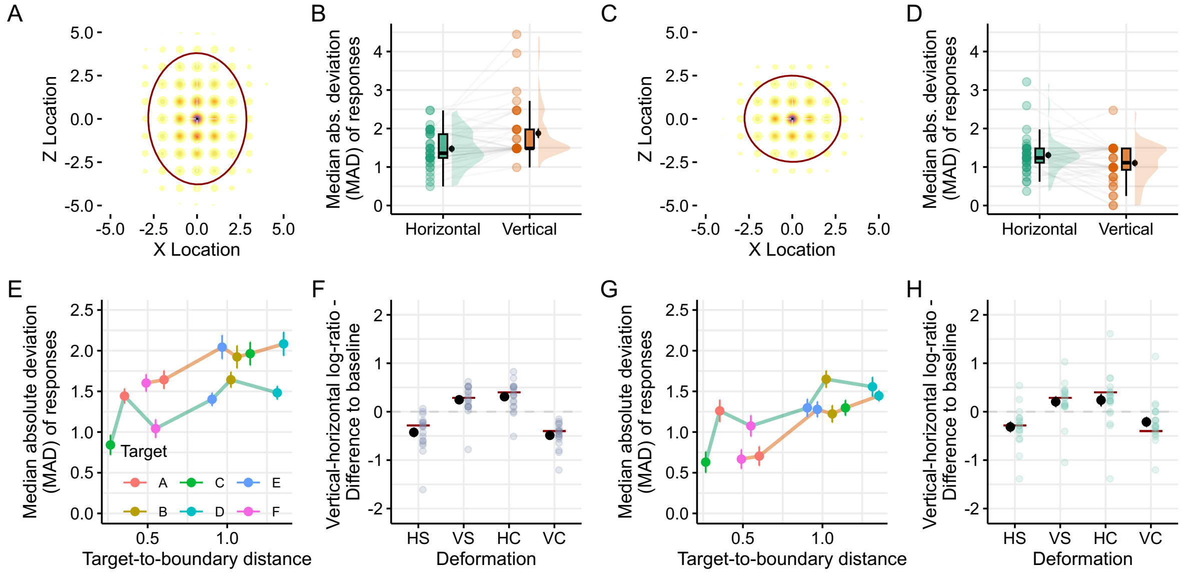

Figure S6: Replication of behavioral findings (cf. Figure 2) based on simulated data from the boundary-proximity model. A-B, E-F: Flying group. C-D, G-H: Walking group. **A, C:** Distribution of median-centered simulated replacement responses in the baseline testing environment, pooled over target locations, reveals larger dispersion along the z-axis in the flying condition (A) and larger dispersion along the x-axis in the walking condition (C). Darker colors reflect higher frequency of values. **B, D:** Median absolute deviation (MAD in environmental bins, 25×25×25 grid) in the baseline for the horizontal (x-, y- axis) and vertical (z-axis) for models individually fitted on flying (B) and walking (D) group data. Violin plots depict the density distribution, boxplots the median and quartiles, mean ± SEM as black dots with error bars, as well as individual model data points (dots) per condition. * P < .05 *** P < .001. **E, G:** MAD for different target locations as a function of target-to-boundary distance, separately computed for the horizontal dimension (green line) and vertical dimension (orange line). For a follow-up analysis of body- and ground-proximity model predictions in the vertically elongated environment, see Fig. S7. **F, H:** Vertical anisotropy indices defined as vertical to horizontal MAD log-ratio for different deformation types (HS/VS: Horizontally/vertically stretched, HC/VC: Horizontally/vertically compressed) relative to baseline. Observed anisotropy indices (dots ± SE) were tested against the expected anisotropy change induced by the 33% deformation (red bars), computed as the log-ratio of the vertical and horizontal deformation size: $\ln(V/H)$; VS: $\ln(1.33/1) = +0.285$; HC: $\ln(1/0.67) = +0.400$; HS: $\ln(1/1.33) = -0.285$; VC: $\ln(0.67/1) = -0.400$).

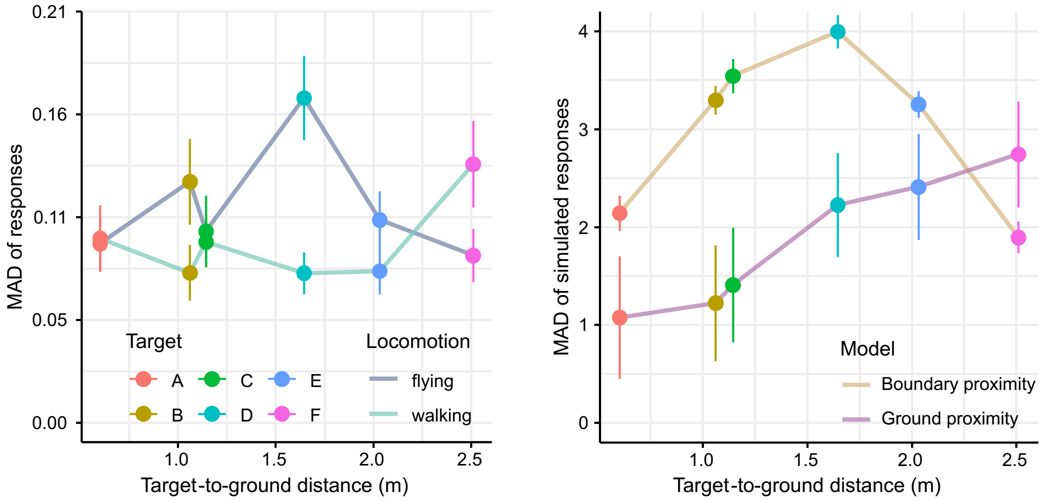

Figure S7: Vertical median absolute response dispersion (MAD) in the vertically enlarged testing environment, shown as a function of target-to-ground distance measured in the baseline environment. The empirical data (*left*) displays the observed MAD (in meters; cf. Fig. 2G/I), contrasting flying (dark blue line) and walking (cyan line) participants. Flying participants exhibited an inverted-U-shaped-like pattern, with the highest location at the center ("D"), indicating an overall boundary-proximity effect. Walking participants show the largest MAD for target location "F" with the highest ground-distance, aligning more closely with the ground-proximity prediction. The simulated model data (*right*) shows the predicted MAD (in environmental bins; cf. Fig. S6). The panel contrasts the boundary-proximity model (golden line) based on parameters fitted on flying participants and the ground-proximity model (purple line) based on parameters fitted on walking participants. Bold dots with error bars depict mean ± SEM.

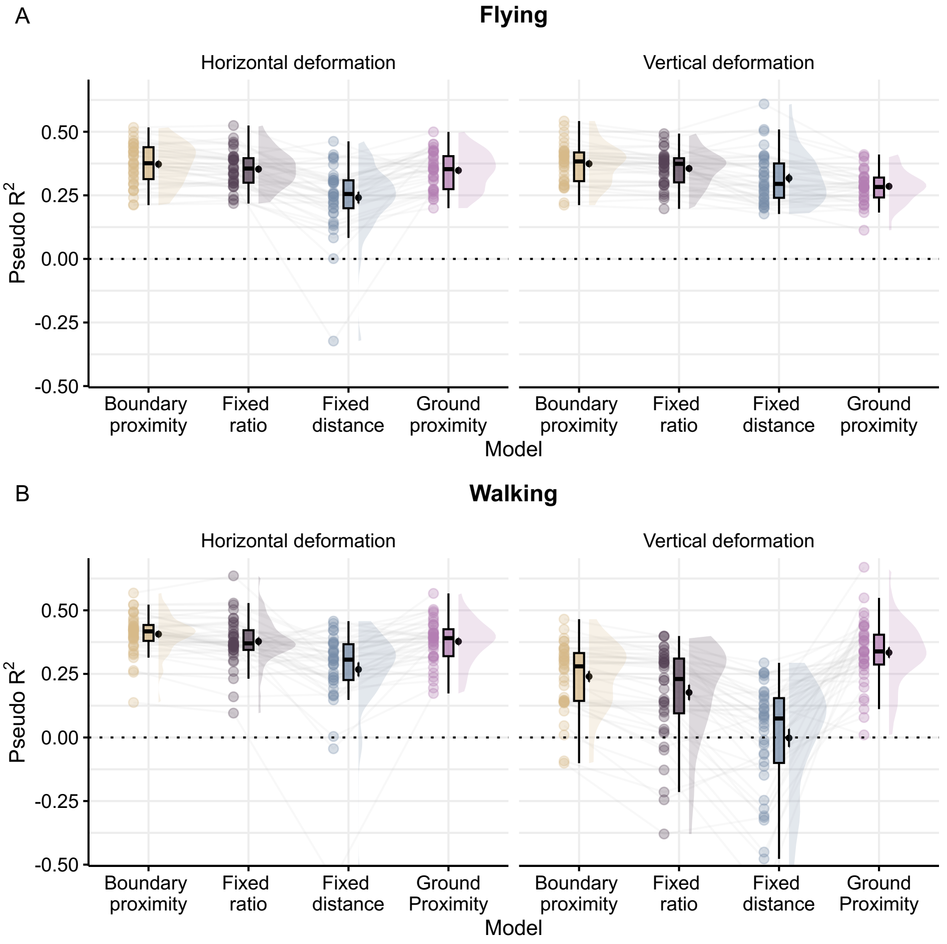

Figure S8: Pseudo-R^2 values (R^2 = 1 - Log-Lik$_{model}$ / Log-Lik$_{chance}$) for each model, separately for horizontal/vertical deformations and for the flying (A) and walking (B) groups. Pseudo-R² values approaching 1 indicate perfect model prediction, values around 0 indicate chance-level performance, and negative values indicate worse-than-chance performance. Violin plots depict the density distribution, boxplots the median and quartiles, black dots with error bars the means ± SEM, and colored dots individual data points per condition. Nearly all tested conditions and models had pseudo-R² values that were significantly greater than zero (one-sample *t*-tests against chance; all P < .001, Bonferroni-corrected for 16 comparisons, $d \geq .938$), except for the fixed-distance model in the vertical deformation condition of the walking group, which did not exceed chance level (t_{39} = -.053, P = 1, d = -.09).

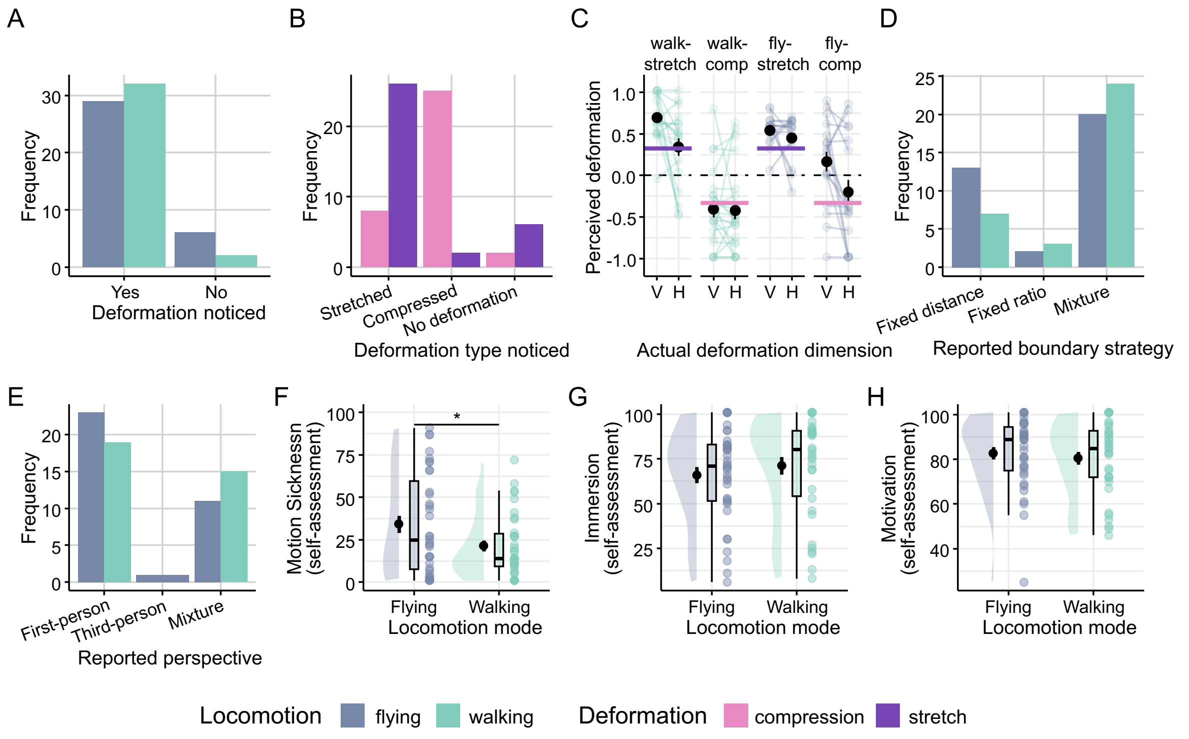

Figure S9. Debriefing summary. A: Number of participants who noticed the deformation of the virtual environment during the test phase. The majority (89.6%) perceived the deformation. Question: "*Did you notice that the virtual environment changed during the test phase?*"; Options: "*Yes*" / "*No*" **B:** Number of participants who correctly identified the type of deformation ['Stretch', 'Compression', or 'None']. 76.6% of participants who perceived the deformation correctly identified the deformation type. Question: "*In which way did the direction change?*"; Options: "*The size of the environment has been increased*", "*The size of the environment has been decreased*". **C:** Group-specific perception of the deformation magnitude (negative values = compression; positive values = stretch; 0 = no change) in the vertical (V) and horizontal (H) dimensions, compared to the actual deformation magnitude (horizontal lines). Most participants tended to correctly estimate horizontal deformations, while those in the walking condition tended to overestimate vertical stretch and those in the flying condition tended to overestimate vertical compression. Question: "*How much did the size change?*"; Options: Bidirectional continuous slider on a visual scale for vertical and horizontal dimension, separately (center = no change relative to baseline, minimum = half the size of baseline, maximum = 2 times baseline). Black dots depict mean ± SEM with error bars, color-coded dots reflect individual data points per condition. **D:** Number of participants who reported using different boundary-related strategies to remember object-locations. The majority (63.08%) of participants reported using a mixture between 'fixed distance' and 'fixed ratio' strategy Question: "*How did you use the walls of the virtual box to remember the location of the objects?";* Options: "*I remembered the edge to the nearest walls*" ['Fixed distance'] / "*I remembered the ratio of opposite walls*" ['Fixed ratio'] / "*A mixture of the two options*" ['Mixture']. **E:** Number of participants who reported using different spatial perspective-taking strategies. 57.14% of participants reported using an egocentric perspective, 1.43% using an allocentric strategy and 41.43% using a mixture strategy, with comparable proportions between locomotion modes. Question: "*What strategy did you use to remember the object-locations?";* Options: "*I tried to remember a snapshot ('first-person perspective')*" ['Egocentric'] / "*I tried to imagine a mental 'map' of the virtual box in order to locate objects on the 'map' ('third-person perspective')*" ['Allocentric'] / "*A mixture of the first two options*" ['Mixture'] **F:** Self-reported motion sickness levels, with flying participants reporting significantly higher motion sickness compared to walking participants (2-sample *t*-test: $t_{58.8} = 2.27$, $P = .027$, $d = .523$). Question: "*Did you feel dizzy or nauseous while performing the task";* Options: Continues slider on a visual scale from "not at all" to "very much". Violin plots depict

the density distribution, boxplots the median and quartiles, mean ± SEM as black dots with error bars, as well as individual data points (dots) per condition. * $P < .05$. **G:** Self-reported immersion levels, with no significant differences between the flying and walking groups (2-sample *t*-test: $t_{75} = -.572$, $P = .569$, $d = -.130$). Question: *"How much did you feel that you were in the 'here and now' while performing the task?";* Options: Continues slider on a visual scale from "not at all" to "very much". **H:** Self-reported motivation levels, with no significant differences between the flying and walking groups (2-sample *t*-test: $t_{68.4} = -.482$, $P = .631$, $d = -.110$). Question: *"How high would you rate your motivation during the experiment?";* Options: Continues slider on a visual scale from "not at all" to "very much".

Table S1. Sample characteristics

Variable	Group 1 (n=20)	Group 2 (n=18)	Group 3 (n=20)	Group 4 (n=19)	Full sample (N=77)	*P*-value
**Gender**						
Female	9 (45%)	10 (55.6%)	12 (60%)	9 (47.4%)	40 (51.9%)	.780
Male	11 (55%)	8 (44.4%)	7 (35%)	10 (52.6%)	36 (46.8%)	.611
Non-binary	0 (0%)	0 (0%)	1 (5%)	0 (0%)	1 (1.3%)	1
**Age**						.408
18-23 years	6 (30%)	9 (50%)	3 (15%)	4 (21.1%)	22 (28.6%)	.106
24-29 years	10 (50%)	5 (27.8%)	12 (60%)	12 (63.2%)	39 (50.6%)	.132
30-35 years	4 (20%)	4 (22.2%)	5 (25%)	3 (15.8%)	16 (20.8%)	.945
**Education**						
< High school	1 (5%)	4 (22.2%)	0 (0%)	1 (5.3%)	6 (7.8%)	.08
High school	7 (35%)	7 (38.9%)	11 (55%)	10 (52.6%)	35 (45.5%)	.546
Bachelor's	10 (50%)	6 (33.3%)	3 (15%)	4 (21.1%)	23 (29.9%)	.09
Master's	2 (10%)	1 (5.56%)	5 (25%)	4 (21.1%)	12 (15.6%)	.322
**Body measures**						
Weight (kg)	66.2 ± 9.44 (45 - 89)	70.2 ± 10.1 (58 - 85)	71.3 ± 12.4 (52 - 94)	73.9 ± 12.6 (51 - 100)	70.36 ± 11.38 (45 - 100)	.206
Height (m)	1.75 ± .076 (1.65 - 1.87)	1.77 ± .05 (1.69 - 1.85)	1.73 ± .061 (1.63 - 1.84)	1.77 ± .07 (1.65 - 1.88)	1.76 ± .07 (1.63 - 1.88)	.196
Arm span (m)	1.72 ± .099 (1.58 - 1.92)	1.75 ± .07 (1.6 -1.86)	1.72 ± .08 (1.6 - 1.85)	1.75 ± .09 (1.57 - 1.9)	1.74 ± .083 (1.57 - 1.92)	.425
Inseam (m)	.794 ± .046 (.73 - .88)	.817 ± .06 (.74 - .98)	.785 ± .03 (.73 - .83)	.795 ± .04 (.7 - .85)	.797 ± .045 (.7 - .98)	.15
Hip width (m)	.368 ± .028 (.34 - .46)	.374 ± .022 (.33 - .4)	.376 ± .021 (.33 - .41)	.371 ± .025 (.34 - .42)	.372 ± .024 (.33 - .46)	.668

Note. Gender, age, and education: Values depict frequency (percentage) for each group and pooled across groups; *P*-values resulted from Fisher's exact test (two-sided) assessing associations with group membership. Body measures: Values depict mean ± SD (min-max) for each group and pooled across groups. *P*-values resulted from 1-way ANOVAs, each with group membership as a between-subject factor. Group 1 = Walking-Stretch; Group 2 = Flying-Stretch; Group 3 = Walking-Compression; Group 4 = Flying-Compression.

Table S2. Environment characteristics

Attribute	Environment A	Environment B	Environment C	Environment D	Environment E
**Geometric shape**	Cube	Cuboid	Cuboid	Cuboid	Cuboid
**Deformation dimension**	-	Horizontal (X)	Vertical (Z)	Horizontal (X)	Vertical (Z)
**Deformation type**	-	Stretch	Stretch	Compression	Compression
**Group**	All	1, 2	1, 2	3, 4	3, 4
**Phase**	Training & Test	Test	Test	Test	Test
**X/length (m)**	3	3.99	3	2.01	3
**Y/width (m)**	3	3	3	3	3
**Z/height (m)**	3	3	3.99	3	2.01
**Re-scaling (m)**	0	.99 (33%)	.99 (33%)	.99 (33%)	.99 (33%)
